# Tumor Necrosis Factor Alpha and Insulin-Like Growth Factor 1 Induced Modifications of the Gene Expression Kinetics of Differentiating Skeletal Muscle Cells

**DOI:** 10.1371/journal.pone.0139520

**Published:** 2015-10-08

**Authors:** Swanhild U. Meyer, Stefan Krebs, Christian Thirion, Helmut Blum, Sabine Krause, Michael W. Pfaffl

**Affiliations:** 1 Physiology Weihenstephan, ZIEL Research Center for Nutrition and Food Sciences, Technische Universität München, Freising, Germany; 2 Laboratory for Functional Genome Analysis (LAFUGA), Gene Center, University of Munich, Ludwig-Maximilians-Universität München, München, Germany; 3 SIRION Biotech GmbH, Martinsried, Germany; 4 Friedrich-Baur-Institute, Department of Neurology, Ludwig-Maximilians-Universität München, München, Germany; University of Minnesota Medical School, UNITED STATES

## Abstract

**Introduction:**

TNF-α levels are increased during muscle wasting and chronic muscle degeneration and regeneration processes, which are characteristic for primary muscle disorders. Pathologically increased TNF-α levels have a negative effect on muscle cell differentiation efficiency, while IGF1 can have a positive effect; therefore, we intended to elucidate the impact of TNF-α and IGF1 on gene expression during the early stages of skeletal muscle cell differentiation.

**Methodology/Principal Findings:**

This study presents gene expression data of the murine skeletal muscle cells PMI28 during myogenic differentiation or differentiation with TNF-α or IGF1 exposure at 0 h, 4 h, 12 h, 24 h, and 72 h after induction. Our study detected significant coregulation of gene sets involved in myoblast differentiation or in the response to TNF-α. Gene expression data revealed a time- and treatment-dependent regulation of signaling pathways, which are prominent in myogenic differentiation. We identified enrichment of pathways, which have not been specifically linked to myoblast differentiation such as doublecortin-like kinase pathway associations as well as enrichment of specific semaphorin isoforms. Moreover to the best of our knowledge, this is the first description of a specific inverse regulation of the following genes in myoblast differentiation and response to TNF-α: Aknad1, Cmbl, Sepp1, Ndst4, Tecrl, Unc13c, Spats2l, Lix1, Csdc2, Cpa1, Parm1, Serpinb2, Aspn, Fibin, Slc40a1, Nrk, and Mybpc1. We identified a gene subset (Nfkbia, Nfkb2, Mmp9, Mef2c, Gpx, and Pgam2), which is robustly regulated by TNF-α across independent myogenic differentiation studies.

**Conclusions:**

This is the largest dataset revealing the impact of TNF-α or IGF1 treatment on gene expression kinetics of early *in vitro* skeletal myoblast differentiation. We identified novel mRNAs, which have not yet been associated with skeletal muscle differentiation or response to TNF-α. Results of this study may facilitate the understanding of transcriptomic networks underlying inhibited muscle differentiation in inflammatory diseases.

## Introduction

**Fig 1:**
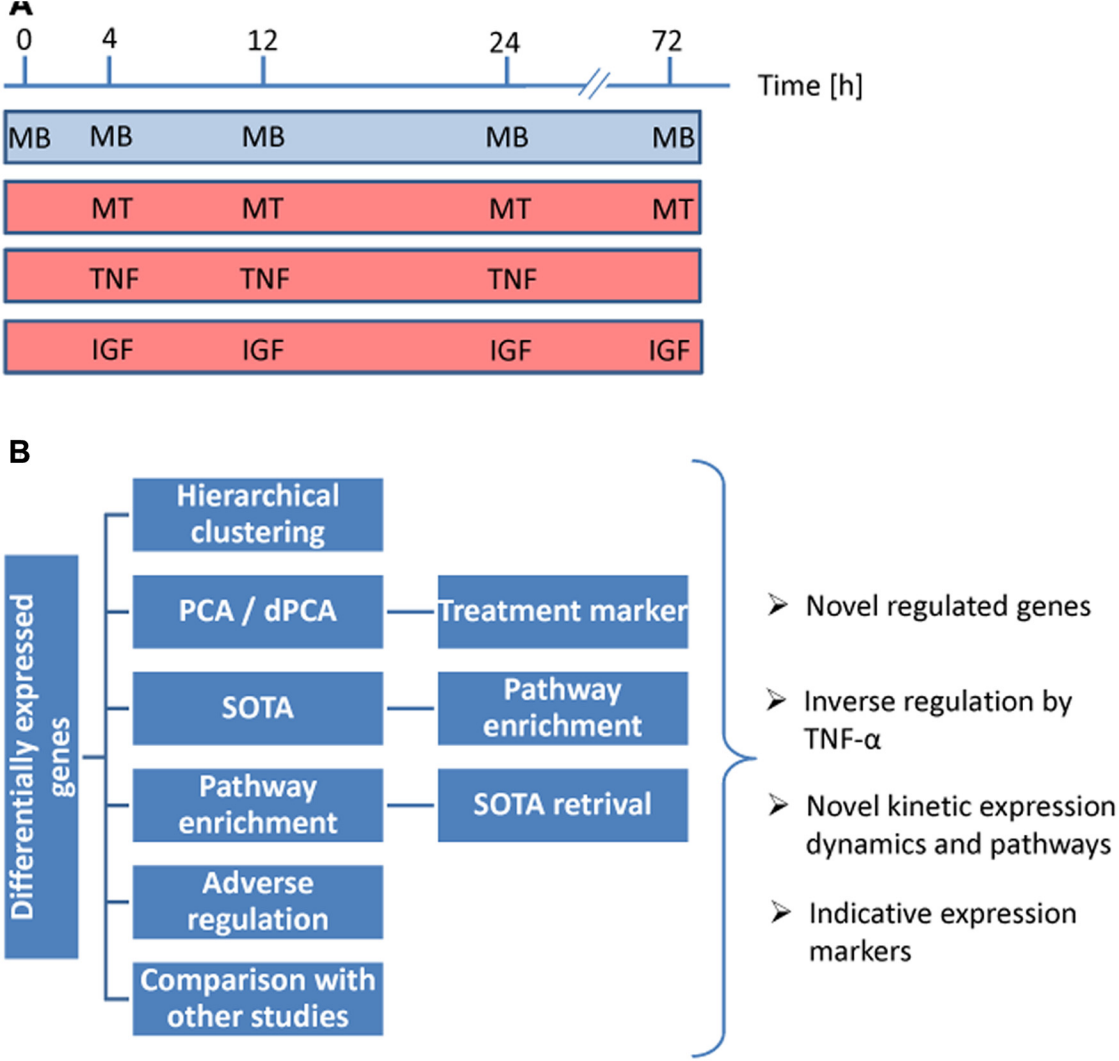
Experimental set up and analyses of expression profiling data. (A) Schematic overview of sampling. MB: myoblasts cultured in growth medium. MT: myotubes cultured in differentiation medium. TNF: myotubes cultured in differentiation medium with TNF-α. IGF: myotubes cultured in differentiation medium with IGF-1. (B) Gene expression data were analyzed by hierarchical clustering, principal component analysis (PCA), and dynamic PCA among others. Moreover, the profiling data were clustered by applying the self-organized tree algorithm (SOTA) as well as pathway association enrichment analyses. Furthermore, genes adversely regulated by differentiation and TNF-α were identified. Finally, TNF-α-regulated genes were compared with results of other studies. The study identified novel insights into the gene expression mechanisms and kinetics of early skeletal myocyte differentiation and how this is modified by TNF-α.

Myoblast differentiation is a multistep process, which involves proliferation, exit from the cell cycle, migration, alignment, and fusion into multinucleated myotubes [1,2]. This process is mediated by a cascade of changes in gene expression [3] and is essential for muscle repair. Myoblast differentiation can be promoted by growth factors such as IGF1 [4], but it is impaired by elevated concentrations of inflammatory cytokines such as TNF-α [5–7]. IGF1 increases myoblast differentiation via both hyperplasia and hypertrophy [5]; however, the underlying regulatory mechanisms at the transcriptomic level are poorly understood. The inhibitory effect of inflammatory levels of TNF-α on myoblast differentiation and muscle repair is associated with cachectic muscle wasting [8,9] and several chronic diseases or muscular disorders [10–12]. Moreover, human aging is associated with muscle inflammation susceptibility [13]. The molecular mechanisms leading to inhibition of mybolast differentiation because of elevated TNF-α concentrations are highly complex, involving modulations at the mRNA level [7] as well as epigenetic implications [3], among others. The molecular signaling pathways mediating the inhibitory effect of TNF-α on myogenic differentiation are not yet completely elucidated. To date, cachectic muscle wasting is an incurable complication [14]; however, several therapeutic strategies are currently being investigated to promote skeletal muscle growth and regeneration [15]. Therefore, the current study addressed the mRNA expression kinetics within the first 24 h up to 72 h of differentiation and concomitant response to IGF1 and TNF-α (Fig 1A). Kinetic expression data obtained from the current study and pathway association analyses as well as principal component analyses and the self-organizing tree algorithm-based clustering provide valuable insights into the molecular signaling mechanisms, which mediated the effect of TNF-α and IGF1 (Fig 1B).

## Materials and Methods

### Cell culture

Murine skeletal myoblasts PMI28 [16] were cultured in growth medium containing Ham’s F10 (PAA Laboratories GmbH, Pasching, Austria), supplemented with 20% FCS (Sigma-Aldrich, St. Louis, MO, USA), 2 mM L-glutamine (PAA Laboratories), and 1% Penicillin/Streptomycin (PAA Laboratories). PMI28 myoblasts were seeded on laminin-1 (Sigma-Aldrich)-coated dishes at a density of 1.5 × 10^6^ cells per 10-cm cell culture plate. Differentiation was induced by switching to serum-reduced medium 24 h after seeding. The differentiation medium was composed of Dulbecco’s Modified Eagle Medium with 2% horse serum (Gibco, Life Technologies GmbH, Darmstadt, Germany), 2 mM L-glutamine (PAA Laboratories) and Penicillin (100 I.U./mL)/Streptomycin (100 μg/mL) (PAA Laboratories) with 2 × 10^3^ U/mL murine recombinant TNF-α (Roche Diagnostics, Rotkreuz, Switzerland) or 5 ng/mL murine recombinant IGF1 (Sigma-Aldrich) or carrier. The growth medium as well as the differentiation control and treatment media were replaced twice a day to ensure cytokine and growth factor activity. Murine PMI28 cells were harvested 4 h, 12 h, 24 h, 48 h, or 72 h after the induction of fusion by serum withdrawal for RNA analyses. For mRNA profiling, approximately 2 × 10^6^ cells were harvested in 1.5 mL Trizol (for details, see the section on RNA extraction and quality control). PMI28 myoblasts were propagated and differentiated at 37°C, 80% relative humidity, and 5% CO_2_.

### Western blot analysis

Protein was extracted using the RIPA Lysis Buffer system (Santa Cruz Biotechnology, Dallas, TX, USA) according to the manufacturer’s instructions. The protein concentration was determined by a bicinchoninic acid (BCA) assay. Gel electrophoresis was performed with NuPAGE Bis-Tris 4–12% Gels (Invitrogen, Life Technologies GmbH) using prestained protein plus ladder (Fermentas GmbH, St. Leon-Rot, Germany), 10 μg protein per sample and 1 × NuPAGE MES SDS Running Buffer (Life Technologies GmbH). Proteins were transferred onto a nitrocellulose membrane using an XCell II Blot Module. Blocking was performed by 5% nonfat dry milk in Tris-Buffered Saline with 0.05% Tween 20 (TBST). The primary antibodies Chk1-antibody (G-4) sc-8408 (Santa Cruz Biotechnology), Emi1-antibody (E-19) sc-50928 (Santa Cruz Biotechnology), or Mybl2-antibody PAB18309 (Abnova, Taipei City, Taiwan) were diluted 1:1,000 or 1:500 with 5% nonfat dry milk in TBST. The antibody used as a normalization reference, H3-antibody Histone H3 (D1H2) XP rabbit mAb (#4499) (Cell Signaling Technology, Danvers, MA, USA), was diluted 1:2,000. In addition, we performed a peptide neutralization assay with the Emi1-antibody sc-50928 (Santa Cruz Biotechnology), which was coincubated with a 5-fold molar excess and a 10-fold molar excess of the Emi (E-19) peptide sc-50928 P (Santa Cruz Biotechnology) prior to application on the blot. The protein blots were incubated with the antibody dilutions overnight at 4°C. Blots were washed with TBST and incubated with horseradish peroxidase-conjugated secondary antibodies antimouse IgG-HRP sc-2031 (Santa Cruz Biotechnology), antirabbit IgG-HRP sc2030 (Santa Cruz Biotechnology), or antigoat IgG-HRP sc2020 (Santa Cruz Biotechnology) diluted in blocking solution for 1 h. After washing with TBST, the blots were incubated with Thermo Scientific SuperSignal West Dura Chemiluminescent Substrate (Thermo Scientific, Thermo Fisher Scientific, Waltham, MA USA) according to the manufacturer’s instructions. Images were acquired using the Fusion FX chemiluminescent scanner with auto exposure settings.

### RNA extraction and quality control

The cells were washed with PBS and lysed in Trizol (Invitrogen, Life Technologies GmbH) to harvest approximately 2 × 10^6^ cells per 1.5 mL Trizol. The samples were homogenized by vigorous shaking. A total of 0.3 mL chloroform was added per 1 mL Trizol, and the samples were mixed for 15 s by vigorous shaking. Phase separation was allowed by placing the samples on the bench top for 10 min followed by centrifugation at 12,000 ×*g* for 25 min at 4°C. The upper aqueous phase was transferred to a fresh tube. A total of 0.75 mL isopropanol per 1 mL Trizol was added, thoroughly mixed, and incubated for 10 min and centrifuged at 12,000 ×*g* at 4°C to precipitate the RNA. The RNA pellet was washed with 0.5 mL ethanol per 1 mL Trizol and centrifugation at 12,000 ×*g* for 10 min at 4°C. The supernatant was aspirated and the sediment was air dried for 15 min. Total RNA was dissolved in nuclease-free water and photometrically quantified by NanoDrop 1000 ND-1000 (Peqlab, Erlangen, Germany) measurement. Moreover, approximately 250 ng RNA were analyzed on 1% Agarose gel with a 1-KB marker for overall RNA quality control.

### Gene expression profiling by hybridization microarrays

Analysis of gene expression was performed with GeneChip Mouse Gene 1.0 ST Arrays (Affymetrix, Santa Clara, CA, USA) following the manufacturer’s instructions. Triplicate samples were analyzed for each time point and treatment. A total of 250 ng total RNA were reverse transcribed using the Ambion WT Expression Kit (Ambion, Life Technologies GmbH, Darmstadt, Germany) including the GeneChip Poly-A RNA Control Kit (Affymetrix) according to the manufacturer’s instructions. The cDNA yield and size distribution were determined and the cDNA was then purified, fragmented, labeled and hybridized applying the GeneChip WT Terminal Labeling and Controls Kits (Affymetrix) following the manufacturer’s instructions. For washing and staining steps the GeneChip Hybridization, Wash, and Stain Kit (Affymetrix) was used according to the manufacturer’s instructions. Fluorescence signals were acquired with the AGCC Scan Control Software. Affymetrix CEL files were read, normalized and summarized using the RMA method [17] as implemented in the Affymetrix apt package. Probe sets were retained if they had at least two “detected above background” present calls in at least one experimental group. GeneChip Mouse Gene 1.0 ST Array data were MIAME [18] compliant and was submitted to the ArrayExpress database (www.ebi.ac.uk/arrayexpress) [19], a publicly available repository consistent with the MIAME guidelines. Data are available with the ArrayExpress accession number E-MTAB-3474.

### Reverse transcription of RNA to cDNA for individual expression analysis

Validation of mRNA expression results obtained by microarray profiling was performed by individual reverse transcription, using gene-specific reverse primer and subsequent qPCR analysis. Reverse transcription was performed using 100 ng total RNA for each reaction and the components of the miScript Reverse Transcription Kit (Qiagen, Hilden, Germany) according to the manufacturer’s instructions.

### Individual quantitative PCR analysis

Individual qPCR reactions were performed with the miScript SYBR Green PCR Kit (Qiagen) according to the manufacturer’s instructions in combination with the respective individually designed primers using PrimerBlast [20]. Primers were ordered from Integrated DNA Technologies, Inc. (Coralville, Iowa, USA) with purification grade desalted. qPCR reactions were performed on CFX384^TM^ Real-Time System C1000^TM^ (Bio-Rad Laboratories, Hercules, CA, USA) to enhance throughput and reduce variation due to the 384-well format. qPCR reaction volumes were equimolarly reduced to 10 μL. In addition, VisiBlue qPCR mix colorant (TATAA Biocenter AB, Gothenburg, Sweden) diluted to a 1:150 dilution was added to facilitate visibility during pipetting. The following thermal cycling conditions were applied: activation of HotStarTaq DNA Polymerase at 95°C for 15 min and three-step cycling with denaturation at 94°C for 15 s, annealing at 55°C for 30 s, extension at 70°C for 30 s, and fluorescence data collection run in 40 cycles. After incubation at 95°C for 5 s the melting analysis was performed from 65°C to 95°C with 0.2°C increments of 5 s for each condition. Relative quantification was performed within the CFX384^TM^ Real-Time System Manager (Bio-Rad Laboratories). Normalization of qPCR-based mRNA expression analyses was performed by taking the mean of Rpl21 and Rpl28, two reference genes, which were selected based on Normfinder [21] analyses results of our mRNA profiling data. Primer sequences of self-designed oligonucleotides: Vegfa forward-5′-AACGATGAAGCCCTGGAGTG, reverse-5′-GCAACGCGAGTCTGTGTTTT; Unc5b forward-5′-CATCCGCATTGCCTACTTGC, reverse-5′-GTGTAGTTGGCCGTGTCTGA; Serpine1 forward-5′-CACAGGCACTGCAAAAGGTC, reverse-5′-GGGCTGAGATGACAAAGGCT; Serpinb2 forward-5′-TTCCGTGTGAACTCGCATGA, reverse-5′-TGCGTCCTCAATCTCATCGG; Rrm2 forward-5′-AGCAAAGCTGCGAGGAGAAT, reverse-5′-CAGAGCTTCCCAGTGCTGAA; Plaur forward-5′-CACAAACCTCTGCAACAGGC, reverse-5′-GGACGCACACTCGAGGTAAC; Nr4a2 forward-5′-GCCTAGCTGTTGGGATGGTT, reverse-5′-GTCAGCAAAGCCAGGGATCT; Mybl2 forward-5′-GGGACCATGGACCAAAGAGG, reverse-5′-AACCTCCCGTGTCGACTTTC; Mcm10 forward-5′-GTGAAGGAGCGTGTGGAGAA, reverse-5′-CCGGGTGGCTCTCATCTTTT; Id2 forward-5′-AAAGCCTTCAGTCCGGTGAG, reverse-5′-TCAGATGCCTGCAAGGACAG; Hmga2 forward-5′-GAAAAACGGCCAAGAGGCAG, reverse-5′-CAGTCTCCTGAGCAGGCTTC; Gja1 forward-5′-CTCACGTCCCACGGAGAAAA, reverse-5′-AGTTGGAGATGGTGCTTCCG; Fgf7 forward-5′-CATGCTTCCACCTCGTCTGT, reverse-5′-CACAATTCCAACTGCCACGG; Fbxo5 forward-5′-ACAATAAGGGGGCGTTCCAG, reverse-5′-AACTCATTGTGCCGGCTGTA; Cxcl12 forward-5′-GCTCTGCATCAGTGACGGTA, reverse-5′-TCAGATGCTTGACGTTGGCT; Chek1 forward-5′-ACTGCAATGTTGGCTGGAGA, reverse-5-GGCCTCTTTGCTCCTCTGTT; Cdkn1a forward-5′-GTACTTCCTCTGCCCTGCTG, reverse-5′-AATCTGTCAGGCTGGTCTGC; Ccnd1 forward-5′-CCCTGGAGCCCTTGAAGA AG, reverse-5′-TCATCCGCCTCTGGCATTTT; Bard1 forward-5′-CTGGTATGCCAGCCAGGAAA, reverse-5′-GAAGCACCGTGGGACAGTAA; Rpl21 forward-5′-CCATAAGTGCTACCACGGCA, reverse-5′-GCCCTTCTCTTTGGCTTCCT; Rpl28 forward-5′-CTTCCGCTACAACGGGCTAA, reverse-5′-GTGTCTGATGCTGCTGAGGG. Differential gene expression was calculated using relative quantification [22].

### Statistics

Significant differences between mRNA expressions measured by individual RT-qPCR analyses were determined using a parametric unpaired two-tailed student’s t-test. Differential expression of genes measured by microarray analysis was determined with LIMMA (Linear Models for Microarray Data) [23] using a factorial design with treatment and time-point as factors. Pairwise comparisons were extracted for all combinations of consecutive time points for the same treatment and between all treatments at the same time point. We clustered expression profiles of all samples for all probesets that were significantly different (fdr < 0.01 and log2 fold change > 1) in at least one pairwise comparison with the self-organizing tree algorithm (SOTA) method [24]. Dynamic PCA was performed within GenEx Software (MultiD Analyses AB, Gothenburg, Sweden) comparing myoblasts to the other treatment groups. Genes were filtered on the basis of p values to identify the most relevant genes explaining the observations [25]. Hierarchical clustering and heatmap generation was performed using GenEx Software (MultiD Analyses AB).

### Bioinformatic analysis of data

The Genomatix Pathway System (GePS) within the Genomatix Software Suite (Genomatix Software GmbH, Munich, Germany) uses pathway data from the Pathway Interaction Database [26]. We applied GePS analysis for identifying significant pathway associations and gene ontology terms of input genes derived from our gene expression profiling data.

## Results

### Immediate response to differentiation and TNF-α treatment

**Fig 2:**
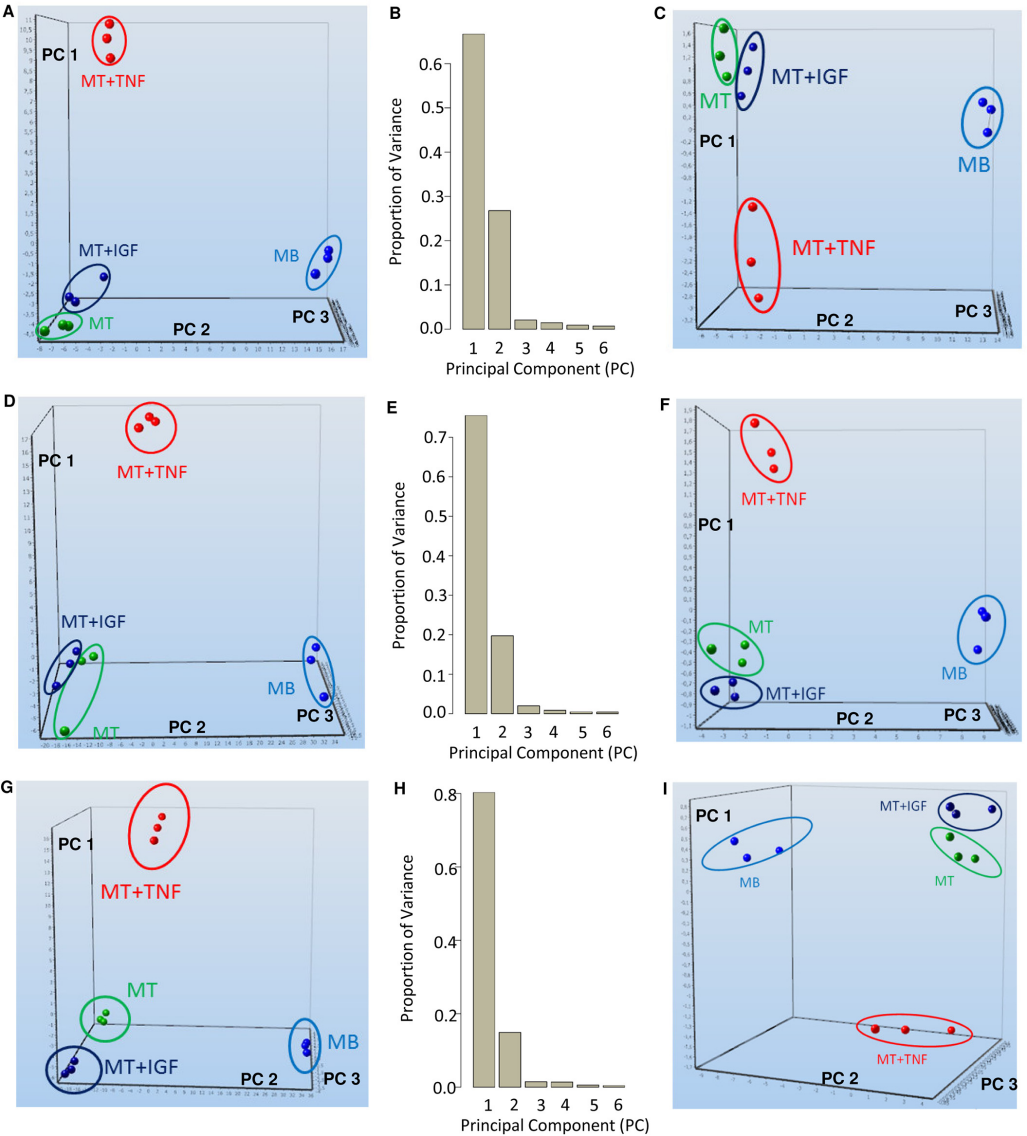
Transcriptomic signatures of myoblast differentiation and TNF-α or IGF1 treatment. Principal component analyses (PCA) of mRNA expression profiling data after (A) 4 h, (D) 12 h, or (G) 24 h of induction of differentiation with TNF-α, IGF1, or control treatment showing nonambiguous genes are depicted. Axes show principal components (PC) 1, PC 2, and PC 3. PCA revealed separation of treatment groups. Light blue indicates myoblasts, green marks myotubes, red distinguishes myotubes treated with TNF-α, whereas dark blue indicates myotubes treated with IGF1. The proportions of variance for the first six components of principal component analysis are depicted for the effect of (B) 4 h, (E) 12 h, and (H) 24 h after induction of differentiation and the respective treatments. Most of the variance is described by PC 1 followed by PC 2 and PC 3. PC 1 explaines most of the variance of myocyte differentiation while PC 2 represented most of the variance induced by TNF-α whereas PC 3 characterized most of the variance caused by IGF1 treatment. Moreover, results from dynamic principal component analyses (dPCA) (group selection myoblasts) are shown for gene expressions (C) 4 h, (F) 12 h, and (I) 24 h after induction of differentiation and treatment. DPCA identified a minimal subset of genes, which could describe the treatment effects (see Table 2) and separate the effects by principal components.

The effect of myoblast differentiation as well as the response to TNF-α or IGF1 exposure modified gene expression patterns, which resulted in separation of treatment groups by hierarchical clustering (S1 Fig) or principal component analysis (PCA) (Fig 2A, 2D and 2G). Genes which separated by principal component analyses (S1 Table) were analyzed for enrichment of pathway associations (Table 1). As early as 4 h after the induction of differentiation and treatment, the gene expression pattern of myoblasts, myotubes, and myotubes treated with TNF-α clearly separate by PCA (Fig 2A, 2D and 2G), whereas the effect of IGF1 became clearly distinct after 24 h (Table 1). Principal component analysis showed that the differentiation effect had the strongest impact on the proportion of variance followed by the effect of TNF-α treatment whereas IGF1 treatment had a minor effect (Fig 2B, 2E and 2H). Dynamic PCA identified a subset of 61 genes after 4 h of treatment (Fig 2C. and Table 2), a subset of 27 genes and two microRNAs after 12-h treatment (Fig 2F, Table 2) as well as a subset of 19 genes and two microRNAs after 24-h treatment (Fig 2I, Table 2); in each case, these were sufficient to separate the treatment groups by principal components. Furthermore, differential gene expression kinetics revealed dynamic time-specific changes of gene regulation (S2 Table). Moreover, certain genes, which were among the 20 most differentially expressed ones, were regulated immediately after the induction (4 h) as well as during very early (12 h) and early (24 h) myoblast differentiation, such as upregulated Adamts5, Ccdc141, Fibin, and downregulated Serpinb2, Gm12824, Npr3, and Sp7 (S2A Table). TNF-α treatment upregulated several genes immediately after induction (4 h), which were still upregulated 12 h as well as 24 h after induction (S2B Table); these included Ccl2, Ccl7, Nfkbie, Tnfaip3, Nfkbia, Bcl3, Vcam1, Slc2a6, Cxcl10, and Mmp9. IGF1 treatment did not result in differentially expressed genes derived from microarray analysis when compared with nontreated myotubes. However, when IGF1-treated samples were compared with TNF-α, several genes were inversely regulated (S2C Table).

**Table 1:** Pathway enrichment analysis of genes separated by principal components.

**Principal component 1 –Effect of myoblast differentiation**				
**Time point**	**Pathway**	**P-value**	**# Genes (observed)**	**List of observed genes**
4h	SPROUTY HOMOLOG (DROSOPHILA)	6.71E-04	3	Etv5, Fgf2, Etv4
4h	**MOTHERS AGAINST DPP HOMOLOG**	8.66E-04	8	Dlx2, Ctgf, Sp7, Foxc2, Smad9, Id1, Smad7, Id2
4h	SEMAPHORIN	1.11E-03	4	Sema6a, Nrp2, Sema3d, Sema5a
4h	ACTIVIN RECEPTOR LIKE KINASE 1	1.21E-03	4	Ctgf, Smad9, Id1, Smad7
4h	**TGF BETA**	1.37E-03	10	Dlx2, C3ar1, Ctgf, Sp7, Foxc2, Dlx1, Smad9, Id1, Smad7, Id2
4h	**RETINOBLASTOMA 1**	2.57E-03	4	Dlx2, Ccnd1, Dlx1, Id2
4h	CYCLIN D1	3.68E-03	5	Dyrk1b, Ccnd1, Ereg, Id1, Id2
4h	CYCLIN D2	4.02E-03	3	Ccnd1, Fgf2, Id2
12h	**NOTCH**	8.80E-03	6	Sox8, Dll1, Bcl6b, Rbm24, Cdkn1c, Heyl
24h	**RYANODINE RECEPTOR**	6.63E-06	6	Ryr1, Casq2, Srl, Dok7, Atp2a1, Trdn
**Principal component 2 –Effect of TNF-α on myoblast differentiation**				
**Time point**	**Pathway**	**P-value**	**# Genes (observed)**	**List of observed genes**
4h	**TUMOR NECROSIS FACTOR (TNF SUPERFAMILY, MEMBER 2)**	2.01E-10	15	Mmp9, Ccl2, Fbxo32, Nfkbia, Cxcl10, Il1rn, Nfkbie, Relb, Vcam1, Fas, Tnip1, Slc40a1, Tnfaip3, Serpinb2, Serpine1
4h	**NF KAPPA B**	2.43E-08	15	Ddx58, Mmp9, Ccl7, Ccl2, Nfkbia, Nfkb2, Cxcl10, Casp4, Nfkbie, Relb, Vcam1, Rrad, Bcl3, Tnip1, Tnfaip3
4h	**CHEMOKINE (C C MOTIF) LIGAND 2**	1.78E-05	3	Ccl7, Ccl2, Cxcl10
4h	TUMOR NECROSIS FACTOR RECEPTOR SUPERFAMILY (FAS, RANK, ETC.)	4.94E-05	8	Ccl2, Nfkbia, Adamts5, Nfkbie, Relb, Epha7, Fas, Tnfaip3
4h	TISSUE INHIBITOR OF METALLOPROTEINASE	9.27E-05	5	Mmp9, Adamts5, Stc1, Fas, Serpine1
4h	IKAPPAB KINASE	1.88E-04	6	Ddx58, Nfkbia, Nfkb2, Nfkbie, Relb, Bcl3
4h	**TNF RECEPTOR ASSOCIATED FACTOR**	2.14E-04	7	Rnd1, Ddx58, Nfkbia, Nfkb2, Fas, Tnip1, Tnfaip3
4h	INTERLEUKIN 18 (INTERFERON GAMMA INDUCING FACTOR)	6.13E-04	4	Ccl2, Il1rn, Vcam1, Fas
4h	PARATHYROID HORMONE	1.00E-03	4	Sp7, Nr4a2, Vdr, Jag1
4h	INTERLEUKIN 6 (INTERFERON, BETA 2)	1.54E-03	6	Ccl2, Maf, Cxcl10, Il1rn, Vcam1, Cp
4h	**INTERLEUKIN 1**	1.95E-03	6	Mmp9, Ccl2, Nfkbia, Cxcl10, Il1rn, Vcam1
4h	INTERLEUKIN 10	3.00E-03	5	Ccl2, Maf, Cxcl10, Il1rn, Fas
4h	MOTHERS AGAINST DPP HOMOLOG	5.76E-03	7	Fbxo32, Sp7, Meox2, Id3, Aspn, Smad6, Serpine1
4h	TGF BETA	7.78E-03	9	Fbxo32, Adamts5, Sp7, Meox2, Id3, Aspn, Dlx1, Smad6, Serpine1
4h	TANK BINDING KINASE 1	8.42E-03	3	Ddx58, Serpinb2, Dtx4
4h	VERY LOW DENSITY LIPOPROTEIN RECEPTOR	9.39E-03	2	Serpinb2, Serpine1
4h	RHO ASSOCIATED, COILED COIL CONTAINING PROTEIN KINASE	9.48E-03	4	Ccl2, Vcam1, D8Ertd82e, Serpine1
12h	**NF KAPPA B**	4.00E-10	23	Mmp9, Cxcl1, Ccl5, Nfkbie, Vcam1, Cd74, Cxcl16, Saa3, Bcl3, Tnfaip3, Id1, Ddx58, Ccl2, Ccl7, Cx3cl1, Nfkbia, Nfkb2, Stap2, Cxcl10, Casp4, Relb, Tnip1, Ptgs2
12h	**TUMOR NECROSIS FACTOR (TNF SUPERFAMILY, MEMBER 2)**	9.00E-09	18	Mmp9, Cxcl1, Ccl5, Il1rn, Nfkbie, Vcam1, Saa3, Fas, Slc40a1, Tnfaip3, Ccl2, Cx3cl1, Nfkbia, Cxcl10, Relb, Tnip1, Ptgs2, Serpinb2
12h	IKAPPAB KINASE	3.93E-06	10	Cxcl1, Ccl5, Nfkbie, Bcl3, Ddx58, Nfkbia, Nfkb2, Stap2, Relb, Ptgs2
12h	**CHEMOKINE (C C MOTIF) LIGAND 2**	1.18E-04	3	Ccl2, Ccl7, Cxcl10
12h	**INTERLEUKIN 1**	1.78E-04	10	Mmp9, Cxcl1, Ccl5, Il1rn, Vcam1, Ccl2, Cx3cl1, Nfkbia, Cxcl10, Ptgs2
12h	TISSUE INHIBITOR OF METALLOPROTEINASE	2.23E-04	6	Mmp9, Sepp1, Cd82, Fas, Timp3, Fgf2
12h	VASCULAR ENDOTHELIAL GROWTH FACTOR	2.46E-04	10	Mmp9, Cxcl1, Sepp1, Angptl4, Timp3, Vegfc, Lix1, Fgf2, Gpr56, Ptgs2
12h	**MYELOID DIFFERENTIATION PRIMARY RESPONSE GENE (88)**	3.08E-04	8	Cxcl1, Ccl5, Saa3, Tnfaip3, Ddx58, Ccl2, Stap2, Cxcl10
12h	**TNF RECEPTOR ASSOCIATED FACTOR**	5.16E-04	9	Rnd1, Fas, Tnfaip3, Ddx58, Nfkbia, Nfkb2, Stap2, Nrk, Tnip1
12h	INTERLEUKIN 10	5.28E-04	8	Il1rn, Cd82, Saa3, Fas, Ccl2, Maf, Cxcl10, Ptgs2
12h	**INTERLEUKIN 6 (INTERFERON, BETA 2)**	5.85E-04	9	Cxcl1, Ccl5, Il1rn, Vcam1, Ccl2, Maf, Cxcl10, Ptgs2, Cp
12h	**INTERLEUKIN 18 (INTERFERON GAMMA INDUCING FACTOR)**	8.20E-04	5	Il1rn, Vcam1, Fas, Ccl2, Cx3cl1
12h	**TOLL LIKE RECEPTOR**	1.28E-03	9	Cxcl1, Ccl5, Saa3, Tnfaip3, Ddx58, Ccl2, Stap2, Cxcl10, Pde4b
12h	T CELL RECEPTOR CD3 COMPLEX	2.19E-03	6	Cd82, Fas, Hcn1, Nedd9, Tnip1, Pde4b
12h	TUMOR NECROSIS FACTOR RECEPTOR SUPERFAMILY (FAS, RANK, ETC.)	3.69E-03	8	Ccl5, Nfkbie, Cxcl16, Fas, Tnfaip3, Ccl2, Nfkbia, Relb
12h	MOTHERS AGAINST DPP HOMOLOG	7.79E-03	10	Id3, Atoh8, Aspn, Smad9, Smad6, Id1, Dlx2, Parm1, Nedd9, Timp3
12h	**TNFRSF1A ASSOCIATED VIA DEATH DOMAIN**	8.35E-03	3	Fas, Tnfaip3, Stap2
12h	MATRIX METALLOPROTEINASE	8.43E-03	8	Mmp9, Ccl5, Enpp2, Ccl2, Cxcl10, Timp3, Ptgs2, Fosl1
12h	F BOX AND WD REPEAT DOMAIN CONTAINING 7	9.53E-03	3	Bcl3, Nfkb2, Myc
12h	INHIBITOR OF GROWTH	9.98E-03	2	Fas, Ptgs2
24h	**NF KAPPA B**	6.19E-09	32	Cd24a, Cxcl16, Tnfaip3, Nfkbia, Cxcl10, Relb, Abcb1b, Mmp9, Cxcl1, Ccl5, Dysf, Cd74, Bcl3, Lbp, Id1, Ccl7, Stap2, Casp4, Tnip1, Ccl2, Cx3cl1, Fmod, Egln3, Nfkbie, Vcam1, Saa3, Olr1, Eda2r, Birc3, Nfkb2, Fabp5, Ptgs2
24h	**TUMOR NECROSIS FACTOR (TNF SUPERFAMILY, MEMBER 2)**	2.86E-06	22	Tnfaip3, Nfkbia, Cxcl10, Relb, Mmp9, Cxcl1, Ccl5, Lor, Lbp, Tnip1, Slc40a1, Ccl2, Selp, Cx3cl1, Serpinb2, C3, Nfkbie, Vcam1, Saa3, Olr1, Birc3, Ptgs2
24h	IKAPPAB KINASE	4.48E-06	14	Rgs4, Nqo1, Nfkbia, Ccnd1, Relb, Irf5, Cxcl1, Ccl5, Bcl3, Stap2, Egln3, Nfkbie, Nfkb2, Ptgs2
24h	**MATRIX METALLOPROTEINASE**	2.94E-04	16	Sema4b, Ecm1, Cxcl10, Srebf2, Mmp9, Ccl5, Wnt5a, Postn, Fosl1, Jam3, Ccl2, Cxcr4, Enpp2, Srpx2, Ptgs2, Zeb1
24h	HYPOXIA INDUCIBLE FACTOR 1, ALPHA SUBUNIT (BASIC HELIX LOOP HELIX TRANSCRIPTION FACTOR)	9.01E-04	12	Slc16a3, Ncoa1, Abcb1b, Cd74, Ndrg1, Cx3cl1, Cxcr4, Egln3, Aqp1, Sp7, Vegfc, Ptgs2
24h	PEROXISOME PROLIFERATOR ACTIVATED RECEPTOR DELTA	9.59E-04	6	Rgs4, Angptl4, Ptgs1, Pla2g4a, Fabp5, Ptgs2
24h	**CHEMOKINE (C C MOTIF) LIGAND 2**	9.71E-04	3	Cxcl10, Ccl7, Ccl2
24h	ENDOTHELIN	1.47E-03	8	Rgs4, Ptgs1, Ret, Cx3cl1, Npr3, Capn6, Gja1, Ptgs2
24h	VASCULAR ENDOTHELIAL GROWTH FACTOR	2.49E-03	13	Angptl4, Fgf2, Mmp9, Cxcl1, Postn, Lix1, Nrp2, Cxcr4, Olr1, Aqp1, Vegfc, Gpr56, Ptgs2
24h	**TNF RECEPTOR ASSOCIATED FACTOR**	2.98E-03	12	Tnfaip3, Nfkbia, Nrk, Irf5, Dysf, Stap2, Tnip1, Rnd1, Casp12, Eda2r, Birc3, Nfkb2
24h	**INTERLEUKIN 1**	4.97E-03	12	Rgs4, Nfkbia, Cxcl10, Mmp9, Cxcl1, Ccl5, Adcy8, Ccl2, Cx3cl1, C3, Vcam1, Ptgs2
24h	CADHERIN 1, TYPE 1, E CADHERIN (EPITHELIAL)	7.45E-03	9	Vwa5a, Ccnd1, Mmp9, Ndrg1, Postn, Gsta4, Gja1, Ptgs2, Zeb1
24h	MYELOID DIFFERENTIATION PRIMARY RESPONSE GENE (88)	8.66E-03	9	Tnfaip3, Cxcl10, Irf5, Cxcl1, Ccl5, Lbp, Stap2, Ccl2, Saa3
**Principal component 3 –Effect of IGF1 on myoblast differentiation**				
**Time point**	**Pathway**	**P-value**	**# Genes (observed)**	**List of observed genes**
4h	non	non	non	non
12h	NUCLEAR FACTOR (ERYTHROID DERIVED 2) LIKE 2	6.43E-04	5	Gsta1, Lor, Gclm, Gsta2, Mir206
12h	VASCULAR ENDOTHELIAL GROWTH FACTOR	2.65E-03	6	Cxcl1, Timp3, Sema6a, Fgf2, Vegfa, Prox1
12h	MATRIX METALLOPROTEINASE	4.69E-03	6	Adam12, Wnt5a, Timp3, Srpx2, Vegfa, Sema5a
12h	V RAF	5.33E-03	4	Pde8a, Ret, Fam83b, Ngf
12h	FIBROBLAST GROWTH FACTOR	1.00E-02	6	Gja1, Dlx1, Fgf2, Vegfa, Ngf, Mir206
24h	CYCLIN A2	2.29E-05	10	Ccne2, Mybl2, Orc1, Mir27b, Cdt1, Cdkn1a, Cdk6, Chek1, Uhrf1, Mcm3
24h	E2F TRANSCRIPTION FACTOR 1	2.39E-04	9	Ccne2, Mybl2, Clspn, Dusp4, Exo1, Cdkn1a, Cdk6, Chek1, Mcm3
24h	CELL DIVISION CYCLE 2, G1 TO S AND G2 TO M	3.84E-04	13	Ccne2, Espl1, Kif18a, Cdt1, Mcm10, Cdkn1a, Cdk6, Hist1h1b, Chek1, Cdca5, Fbxo5, Uhrf1, Mcm3
24h	PEROXISOME PROLIFERATOR ACTIVATED RECEPTOR DELTA	5.58E-04	6	Rgs4, Ptgs1, Nr4a2, Ckm, Fabp5, Ptgs2
24h	RYANODINE RECEPTOR	8.15E-04	6	Tmem38a, Srl, Pvalb, Casq2, Scn5a, Trdn
24h	CADHERIN 1, TYPE 1, E CADHERIN (EPITHELIAL)	1.06E-03	10	Vwa5a, Ezr, Ndrg1, Gsta4, Rrm2, Gja1, Hgf, Fbxo5, Dcn, Ptgs2
24h	HYPOXIA INDUCIBLE FACTOR 1, ALPHA SUBUNIT (BASIC HELIX LOOP HELIX TRANSCRIPTION FACTOR)	1.29E-03	11	Slc16a3, Idh1, Ndrg1, Mir23b, Cx3cl1, Smpx, Vegfa, Cxcr4, Egln3, Vegfc, Ptgs2
24h	CHECKPOINT KINASE 2	2.20E-03	7	Brca1, Clspn, Exo1, Mcm10, Msh3, Cdkn1a, Chek1
24h	ENDOTHELIN	3.45E-03	7	Rgs4, Plcb4, Mylpf, Ptgs1, Cx3cl1, Gja1, Ptgs2
24h	FANCONI ANEMIA COMPLEMENTATION GROUP COMPLEX	3.93E-03	6	Brca1, Clspn, Exo1, Bard1, Chek1, Uhrf1
24h	ATAXIA TELANGIECTASIA AND RAD3 RELATED	4.95E-03	7	Brca1, Clspn, Exo1, Bard1, Cdt1, Chek1, Mcm3
24h	MITOGEN ACTIVATED PROTEIN KINASE	8.06E-03	23	Tnik, Cmklr1, Slc4a4, Dusp4, Dusp9, Fdps, Fosl1, Car2, Cx3cl1, Gsta4, Fam83b, Nid2, Gja1, Adora1, Klhl31, Mc4r, Olr1, C1qtnf3, Nefm, Dusp5, 2810417H13Rik, Etv4, Ptgs2

Signal transduction pathway associations of genes which are separated by principal component analysis after 4-h, 12-h, or 24-h treatment are depicted. Principal component one separates the effect of differentiation, whereas principal component two represents the effect of TNF-α treatment. Finally, principal component three separates the effect of IGF1 treatment versus control myotubes. Pathway enrichment was based on cocitation with a p value cutoff of <0.01. Genes within significantly enriched pathways are listed. Pathways highlighted in bold are retrieved in enrichment analyses of differentially expressed genes which are shown in Table 3.

**Table 2:** Genes describing the treatment effects.

**4 h myoblast differentiation: control, TNF-α or IGF1 treatment**	
**Gene symbol**	**Gene title**
Tmeff2	transmembrane protein with EGF-like and two follistatin-like domains 2
Sdpr	serum deprivation response
Nrp2	neuropilin 2
Fam78b	family with sequence similarity 78, member B
Sh2d1b1	SH2 domain protein 1B1
Pkp1	plakophilin 1
Ctgf	connective tissue growth factor
Etv5	ets variant gene 5
Chst11	carbohydrate sulfotransferase 11
Aldh3a1	aldehyde dehydrogenase family 3, subfamily A1
Etv4	ets variant gene 4 (E1A enhancer binding protein, E1AF)
Lpin1	lipin 1
Id2	inhibitor of DNA binding 2
Pgf	placental growth factor
Itgb8	integrin beta 8
Idi1	isopentenyl-diphosphate delta isomerase
Serpinb6b	serine (or cysteine) peptidase inhibitor, clade B, member 6b
Rbm24	RNA binding motif protein 24
Serpinb1a	serine (or cysteine) peptidase inhibitor, clade B, member 1a
Slc22a23	solute carrier family 22, member 23
Tmem171	transmembrane protein 171
Hmgcs1	3-hydroxy-3-methylglutaryl-Coenzyme A synthase 1
Sema5a	sema domain, seven thrombospondin repeats (type 1 and type 1-like), transmembrane domain (TM) and short cytoplasmic domain, (semaphorin) 5A
Vdr	vitamin D receptor
Sp7	Sp7 transcription factor 7
Etv5	ets variant gene 5
Robo2	roundabout homolog 2 (Drosophila)
Smad7	MAD homolog 7 (Drosophila)
Hbegf	heparin-binding EGF-like growth factor
Sema6a	sema domain, transmembrane domain (TM), and cytoplasmic domain, (semaphorin) 6A
Dusp5	dual specificity phosphatase 5
Syt12	synaptotagmin XII
Dlx1	distal-less homeobox 1
Ak4	adenylate kinase 4
Id1	inhibitor of DNA binding 1
Qrfp	pyroglutamylated RFamide peptide
Idi1	isopentenyl-diphosphate delta isomerase
Dlx2	distal-less homeobox 2
Ccdc141	coiled-coil domain containing 141
Jag1	jagged 1
Fgf2	fibroblast growth factor 2
Hspa4l	heat shock protein 4 like
Smad9	MAD homolog 9 (Drosophila)
Prr9	proline rich 9
Lce1g	late cornified envelope 1G
Lphn2	latrophilin 2
Gm12824	predicted gene 12824
Id3	inhibitor of DNA binding 3
Sema3d	sema domain, immunoglobulin domain (Ig), short basic domain, secreted, (semaphorin) 3D
Lrrc17	leucine rich repeat containing 17
Ereg	epiregulin
Hk2	hexokinase 2
C3ar1	complement component 3a receptor 1
Dyrk1b	dual-specificity tyrosine-(Y)-phosphorylation regulated kinase 1b
Pdlim3	PDZ and LIM domain 3
Cx3cl1	chemokine (C-X3-C motif) ligand 1
Foxc2	forkhead box C2
Sc4mol	sterol-C4-methyl oxidase-like
1600029D21Rik	RIKEN cDNA 1600029D21 gene
Ubash3b	ubiquitin associated and SH3 domain containing, B
Smad6	MAD homolog 6 (Drosophila)
**12 h myoblast differentiation: control, TNF-α or IGF1 treatment**	
**Gene symbol**	**Gene title**
miR-206	microRNA-206-3p
miR-133b	microRNA-133b-3p
Myog	myogenin
Selp	selectin, platelet
Olfml2b	olfactomedin-like 2B
Lgr6	leucine-rich repeat-containing G protein-coupled receptor 6
Fabp7	fatty acid binding protein 7, brain
Idi1	isopentenyl-diphosphate delta isomerase
Rbm24	RNA binding motif protein 24
Stc1	stanniocalcin 1
Sntb1	syntrophin, basic 1
Sp7	Sp7 transcription factor 7
Adamts5	a disintegrin-like and metallopeptidase (reprolysin type) with thrombospondin type 1 motif, 5 (aggrecanase-2)
Dll1	delta-like 1 (Drosophila)
Dtx4	deltex 4 homolog (Drosophila)
Slc24a3	solute carrier family 24 (sodium/potassium/calcium exchanger), member 3
Id1	inhibitor of DNA binding 1
Idi1	isopentenyl-diphosphate delta isomerase
Dlx2	distal-less homeobox 2
Ttn	titin
Ccdc141	coiled-coil domain containing 141
Fibin	fin bud initiation factor homolog (zebrafish)
Slc7a11	solute carrier family 7 (cationic amino acid transporter, y+ system), member 11
Gm12824	predicted gene 12824
Car8	carbonic anhydrase 8
Dync1i1	dynein cytoplasmic 1 intermediate chain 1
Cpa1	carboxypeptidase A1
Cdkn1c	cyclin-dependent kinase inhibitor 1C (P57)
Sc4mol	sterol-C4-methyl oxidase-like
**24 h myoblast differentiation: control, TNF-α or IGF1 treatment**	
**Gene symbol**	**Gene title**
Car8	carbonic anhydrase 8
Ccdc141	coiled-coil domain containing 141
Cdkn1c	cyclin-dependent kinase inhibitor 1C (P57)
Cpa1	carboxypeptidase A1
Ctrb1	chymotrypsinogen B1
Dlx2	distal-less homeobox 2
Dtx4	deltex 4 homolog (Drosophila)
Dync1i1	dynein cytoplasmic 1 intermediate chain 1
Erbb3	v-erb-b2 erythroblastic leukemia viral oncogene homolog 3 (avian)
Fabp7	fatty acid binding protein 7, brain
Hfe2	hemochromatosis type 2 (juvenile) (human homolog)
Lgr6	leucine-rich repeat-containing G protein-coupled receptor 6
miR-133b	microRNA-133b-3p
miR-206	microRNA-206-3p
Myog	myogenin
Olfml2b	olfactomedin-like 2B
Slc24a3	solute carrier family 24 (sodium/potassium/calcium exchanger), member 3
Slc7a11	solute carrier family 7 (cationic amino acid transporter, y+ system), member 11
Sntb1	syntrophin, basic 1
Stc1	stanniocalcin 1
Ttn	titin

Nonambigous genes identified by dynamic principal component analysis (group selection myoblasts) of gene expression profiling data after 4 h, 12 h, or 24 h of differentiation or TNF-α or IGF1 treatment identified genes that were sufficient for separation of treatment groups by principal components as shown in Fig 2C, 2F and 2I.

### Coregulation of gene sets

**Fig 3:**
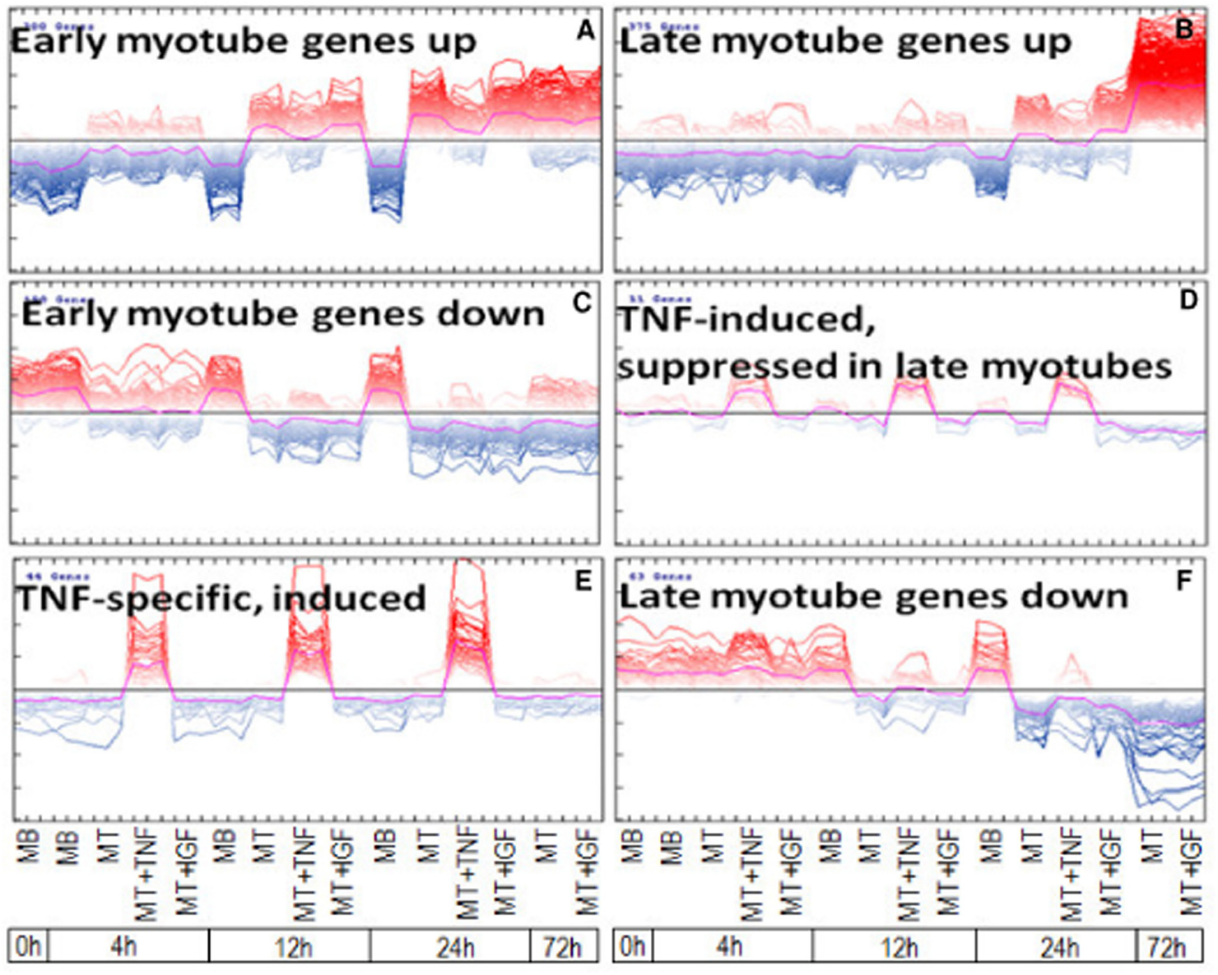
Coregulation of gene sets during myogenic differentiation as well as TNF-α and IGF1 treatment. Self-organizing tree algorithm (SOTA) analysis of gene expression data within the first 72 h of differentiation as well as TNF-α and IGF1 treatment revealed the following clusters of gene sets: (A) cluster A contained 335 genes upregulated during very early differentiation and (B) cluster B comprised 351 genes upregulated during later differentiation. (C) Genes totaling 172, which were downregulated during very early differentiation, were summarized by cluster C. (D) Genes induced by TNF-α but suppressed during late myotubes were visualized by cluster C implying eight genes. (E) Cluster E included 40 genes specifically induced by TNF-α. (F) Forty genes downregulated later during differentiation were represented by cluster F. Gene identities and signal transduction pathway associations of genes within the individual SOTA clusters are depicted in S4 Table. Furthermore, clusters G, H, and I bear the minority of genes and are depicted in S2 Fig.

Self-organizing tree algorithm (SOTA) analyses of gene expression over time (0 h, 4 h, 12 h, 24 h, and 72 h) revealed significant coregulation of gene sets in response to the induction of differentiation and confirmed the immediate response to TNF-α or IGF1 treatment (Fig 3). Clustered cohorts of the gene expression pattern showed a distinct shift in expression levels as early as 4 h after induction of differentiation and TNF-α or IGF1 treatment (Fig 3). The majority of differentially expressed genes fitted in one of the six clusters as shown in Fig 3. The other three clusters (S2 Fig) and corresponding gene lists of all clusters (S3 Table) are shown in the supporting information section. The data collected from the current study suggests that >80% of the differentially expressed genes clustering in cohorts are assigned to three SOTA clusters: cluster A, which includes genes upregulated during very early differentiation (Fig 3A); cluster B, which represents genes upregulated during late differentiation (Fig 3B); and cluster C, which visualized cohorts of genes downregulated as early as 4 h after induction of differentiation (Fig 3C). We examined whether gene expression transcripts with similar regulation also demonstrate related biological implications. Analysis of signal transduction pathway associations and gene ontology annotation class “biological processes” (S4 Table) demonstrate that pathways such as cyclin G1 and semaphorin pathway were enriched in cluster A (early myotubes genes up). Cluster B (late myotube genes up) was enriched for genes with a function in the ryanodine receptor and calcineurin pathway for example, whereas cluster C (early myotube genes down) showed enrichment of genes e.g. involved in dual-specific phosphatase and fibroblast growth factor pathway as well as TGFbeta signaling. Genes with a function in, for example, the cyclin-dependent kinase inhibitor 2 pathway were enriched in cluster D (TNF induced, suppressed in late myotubes), while cluster E (specifically induced by TNF) overrepresented genes with a function in pathways such as NFkappaB and tumor necrosis factor. Finally, cluster F (late myotubes genes down) was enriched for pathways such as nuclear factor (erythroid derived 2) like 2, tumor protein p 53, and other cell cycle-related pathways.

### Specific signaling pathway regulation during myoblast differentiation and TNF-α response

Signal transduction pathway associations were enriched in a time-dependent manner (Table 3) for the effect of myoblast differentiation. Immediately after the induction of differentiation (4-h differentiation), mothers against DPP homolog (Smad) and TGFbeta pathway associations were significantly enriched amongst others (Table 3). During very early differentiation (12-h differentiation), signaling pathways such as mothers against DPP homolog, notch, and semaphorin were enriched (Table 3). After 24-h differentiation (early differentiation) enrichment analysis of pathway associations revealed involvement of cyclin A2, ryanodine receptor, and E2F transcription factor 1 pathway among others (Table 3). For example, pathways related to TGFbeta or SMAD signaling (Table 3) were additionally allocated to SOTA cluster C. However, part of the enriched signal transduction pathway association included genes which were not similarly regulated during myoblast differentiation as the respective pathway associations were not retrieved in SOTA clusters.

**Table 3:** Pathway enrichment analysis of differentially expressed genes.

**Myoblast differentiation**					
**Time point**	**Pathway**	**P-value**	**# Genes (observed)**	**List of observed genes**	**SOTA cluster**
4 h	**MOTHERS AGAINST DPP HOMOLOG**	1.16E-05	12	Id3, Foxc2, Aspn, Smad9, Smad6, Id1, Smad7, Id2, Dlx2, Ctgf, Sp7, Serpine1	C
4 h	**TGF BETA**	3.08E-05	15	Fbxo32, Foxc2, Aspn, Itgb8, Dlx1, Smad9, Smad6, Id1, Smad7, Id2, Dlx2, Ctgf, Adamts5, Sp7, Serpine1	C
4 h	PARATHYROID HORMONE RELATED PROTEIN	7.05E-04	5	Igfbp5, Ctgf, Sp7, Nr4a2, Jag1	
4 h	HAIRY AND ENHANCER OF SPLIT 1	6.22E-03	3	Dll1, Ctgf, Jag1	
4 h	**RETINOBLASTOMA 1**	6.42E-03	4	Dlx1, Id2, Dlx2, Ccnd1	
4 h	VERY LOW DENSITY LIPOPROTEIN RECEPTOR	7.48E-03	2	Serpinb2, Serpine1	C
4 h	LYMPHOID ENHANCER BINDING FACTOR 1 (TCF/LEF)	7.62E-03	3	Dll1, Vdr, Ccnd1	
4 h	SEMAPHORIN	9.75E-03	3	Nrp2, Sema6a, Sema5a	A
12 h	MOTHERS AGAINST DPP HOMOLOG	2.32E-05	289	Atoh8, Aspn, Smad9, Smad7, Id2, Dlx2, Cdkn1c, Timp3, Nedd9, Scn5a, Id3, Foxc2, Hfe2, Id1, Smad6, Ctgf, Sp7, Mir206	C
12 h	**NOTCH**	1.96E-03	251	Sox8, Dll1, Bcl6b, Dlx2, Heyl, Myc, Fabp7, Adam12, Id3, Dlx1, Id1, Mir206, Prox1	
12 h	SEMAPHORIN	2.71E-03	47	Sema6d, Sema6a, Vegfa, Sema5a, Sema3c	B
12 h	CADHERIN 2, TYPE 1, N CADHERIN (NEURONAL)	4.54E-03	33	Fgfr4, Fgf2, Gja1, Itga6	
12 h	TGF BETA	5.42E-03	490	Fbxo32, Atoh8, Aspn, Smad9, Smad7, Id2, Dlx2, Adamts5, Timp3, Nedd9, Scn5a, Adam12, Foxc2, Hfe2, Dlx1, Id1, Smad6, Ctgf, Sp7	C
12 h	FIBROBLAST GROWTH FACTOR	6.13E-03	254	Bcl6b, Dlx2, Myog, Fgfr4, Fgf2, Vegfa, Ngf, Gja1, Spry1, Dlx1, Mir206, Prox1	C
12 h	NERVE GROWTH FACTOR	7.98E-03	111	Fgf2, Ngf, Alcam, Dlx1, Nefm, Id1, Ret	
24 h	CYCLIN A2	5.87E-04	49	Ncoa1, Ccnd1, Rb1, Mybl2, Cdt1, Cdkn1a, Chek1, Uhrf1	
24 h	**RYANODINE RECEPTOR**	6.50E-04	28	Ryr1, Casq1, Srl, Ryr3, Casq2, Trdn	B
24 h	E2F TRANSCRIPTION FACTOR 1	8.84E-04	52	Cdkn1c, Ccnd1, Myc, Rb1, Mybl2, Dusp4, Cdkn1a, Chek1	
24 h	CYCLIN E	1.52E-03	44	Cdkn1c, Ccnd1, Myc, Rb1, Cdkn1a, Chek1, Mcm3	
24 h	PEROXISOME PROLIFERATOR ACTIVATED RECEPTOR DELTA	3.09E-03	26	Rgs4, Bcl6, Nr4a2, Pla2g4a, Ptgs2	C
24 h	CYCLIN DEPENDENT KINASE	3.73E-03	210	Efna5, Ncoa1, Msln, Id2, Cdkn1c, Ccnd1, Myc, Parvb, Rb1, Mybl2, Cdt1, Myog, Cdkn1a, Hist1h1b, Chek1, Nefm, Mcm3	
24 h	NOTCH	4.65E-03	251	Sox8, Bcl6b, Heyl, Myc, Fabp7, Asb2, Id3, Adcy8, Dlx1, Id1, Mfap5, Mir206, Dll1, Dlx2, Mir23b, Smpx, Neurl1a, Prox1, Zeb1	
24 h	HYPOXIA INDUCIBLE FACTOR 1, ALPHA SUBUNIT (BASIC HELIX LOOP HELIX TRANSCRIPTION FACTOR)	5.87E-03	100	Slc16a3, Ncoa1, Idh1, Ndrg1, Id1, Mir23b, Abcc1, Vegfa, Egln3, Ptgs2	C
24 h	CYCLIN D2	6.79E-03	31	Bcl6, Ccnd1, Myc, Fgf2, Rb1	
24 h	CYCLIN DEPENDENT KINASE INHIBITOR 2	7.01E-03	20	Cdkn1c, Ccnd1, Rb1, Cdkn1a	D
24 h	SEMAPHORIN	9.91E-03	47	Sema6d, Sema6a, Nrp2, Vegfa, Sema5a, Sema3c	A
24 h	MOTHERS AGAINST DPP HOMOLOG	9.93E-03	289	Smad9, Smad7, Id2, Cdkn1c, Id3, Foxc2, Smad6, Id1, Mir206, Cilp, Atoh8, Aspn, Dlx2, Hmga2, Mir23b, Scn5a, Hfe2, Sp7, Dcn, Zeb1	C
**Myoblast differentiation with TNF-α treatment**					
**Time point**	**Pathway**	**P-value**	**# Genes (observed)**	**List of observed genes**	**SOTA cluster**
4 h	**TUMOR NECROSIS FACTOR (TNF SUPERFAMILY, MEMBER 2)**	2.92E-10	12	Mmp9, Ccl2, Fas, Nfkbia, Tnip1, Slc40a1, Tnfaip3, Cxcl10, Serpinb2, Nfkbie, Relb, Vcam1	E
4 h	**NF KAPPA B**	5.89E-07	11	Mmp9, Ccl2, Bcl3, Nfkbia, Nfkb2, Tnfaip3, Cxcl10, Nfkbie, Relb, Vcam1, Mcc	E
4 h	**CHEMOKINE (C C MOTIF) LIGAND 2**	1.54E-05	5	Ccl2, Ccl7, Nfkbia, Cxcl10, Vcam1	E
4 h	**INTERLEUKIN 1**	7.36E-04	5	Mmp9, Ccl2, Nfkbia, Cxcl10, Vcam1	E
4 h	RECEPTOR ACTIVATOR OF NUCLEAR FACTOR KAPPA B LIGAND	9.58E-04	5	Fas, Nfkbia, Tnfaip3, Nfkbie, Relb	
4 h	CD40 LIGAND	1.06E-03	3	Fas, Nfkbia, Tnip1	
4 h	INTERLEUKIN 18 (INTERFERON GAMMA INDUCING FACTOR)	1.13E-03	3	Ccl2, Fas, Vcam1	E
4 h	**TNF RECEPTOR ASSOCIATED FACTOR**	2.56E-03	4	Fas, Nfkbia, Nfkb2, Tnfaip3	E
12 h	**NF KAPPA B**	1.28E-12	18	Ddx58, Mmp9, Ccl2, Cxcl1, Nfkbia, Nfkb2, Stap2, Ccl5, Cxcl10, Nfkbie, Relb, Vcam1, Cd74, Mcc, Saa3, Bcl3, Tnfaip3, Capn6	E
12 h	**TUMOR NECROSIS FACTOR (TNF SUPERFAMILY, MEMBER 2)**	4.29E-12	15	Mmp9, Ccl2, Cxcl1, Nfkbia, Ccl5, Cxcl10, Nfkbie, Relb, Vcam1, Saa3, Fas, Tnip1, Slc40a1, Tnfaip3, Serpinb2	E
12 h	**CHEMOKINE (C C MOTIF) LIGAND 2**	1.74E-07	7	Ccl7, Ccl2, Cxcl1, Nfkbia, Ccl5, Cxcl10, Vcam1	C; E
12 h	**TOLL LIKE RECEPTOR**	2.08E-05	7	Ddx58, Cxcl1, Stap2, Ccl5, Cxcl10, Saa3, Tnfaip3	
12 h	**TNF RECEPTOR ASSOCIATED FACTOR**	1.02E-04	6	Ddx58, Nfkbia, Nfkb2, Stap2, Fas, Tnfaip3	E
12 h	**INTERLEUKIN 1**	3.70E-04	6	Mmp9, Ccl2, Cxcl1, Nfkbia, Cxcl10, Vcam1	E
12 h	**MYELOID DIFFERENTIATION PRIMARY RESPONSE GENE (88)**	3.80E-04	5	Ddx58, Cxcl1, Stap2, Ccl5, Cxcl10	E
12 h	RECEPTOR ACTIVATOR OF NUCLEAR FACTOR KAPPA B LIGAND	5.05E-04	6	Nfkbia, Ccl5, Nfkbie, Relb, Fas, Tnfaip3	
12 h	**INTERLEUKIN 6 (INTERFERON, BETA 2)**	1.16E-03	5	Ccl2, Cxcl1, Ccl5, Cxcl10, Cp	E
12 h	CD40 LIGAND	2.40E-03	3	Nfkbia, Fas, Tnip1	
12 h	**INTERLEUKIN 18 (INTERFERON GAMMA INDUCING FACTOR)**	2.55E-03	3	Ccl2, Vcam1, Fas	E
12 h	NUCLEOTIDE OLIGOMERIZATION DOMAIN/CASPASE RECRUITMENT DOMAIN PROTEIN FAMILY	7.86E-03	3	Ddx58, Ccl5, Tnfaip3	
12 h	**TNFRSF1A ASSOCIATED VIA DEATH DOMAIN**	7.93E-03	2	Stap2, Fas	
24 h	**TUMOR NECROSIS FACTOR (TNF SUPERFAMILY, MEMBER 2)**	3.25E-10	16	Mmp9, Cxcl1, Ccl5, Nfkbie, Vcam1, Saa3, Lbp, Slc40a1, Tnfaip3, Ccl2, Birc3, Nfkbia, Cxcl10, Relb, Tnip1, Serpinb2	E
24 h	**NF KAPPA B**	5.79E-10	19	Mmp9, Cxcl1, Ccl5, Nfkbie, Vcam1, Cd74, Saa3, Bcl3, Tnfaip3, Ccl2, Birc3, Nfkbia, Nfkb2, Stap2, Cxcl10, Relb, Mcc, Abcb1b, Capn6	E
24 h	**CHEMOKINE (C C MOTIF) LIGAND 2**	1.03E-08	9	Cxcr4, Cxcl1, Ccl5, Vcam1, Ccl7, Ccl2, Nfkbia, Cxcl10, Abcb1b	C; E
24 h	TOLL LIKE RECEPTOR	2.92E-04	7	Cxcl1, Ccl5, Saa3, Lbp, Tnfaip3, Stap2, Cxcl10	E
24 h	**INTERLEUKIN 1**	3.15E-03	6	Mmp9, Cxcl1, Vcam1, Ccl2, Nfkbia, Cxcl10	E
24 h	**MATRIX METALLOPROTEINASE**	4.08E-03	6	Mmp9, Cxcr4, Ccl5, Enpp2, Postn, Adamts5	C
24 h	**TNF RECEPTOR ASSOCIATED FACTOR**	5.66E-03	5	Tnfaip3, Birc3, Nfkbia, Nfkb2, Stap2	
24 h	INTERLEUKIN 6 (INTERFERON, BETA 2)	6.82E-03	5	Cxcl1, Ccl5, Ccl2, Cxcl10, Cp	E
**Myoblast differentiation with IGF1 treatment**					
**Time point**	**Pathway**	**P-value**	**# Genes (observed)**	**List of observed genes**	**SOTA cluster**
4 h	TUMOR NECROSIS FACTOR (TNF SUPERFAMILY, MEMBER 2)	1.58E-10	12	Mmp9, Ccl2, Fas, Nfkbia, Tnip1, Tnfaip3, Cxcl10, Serpinb2, Il1rn, Nfkbie, Relb, Vcam1	E
4 h	NF KAPPA B	2.96E-08	12	Ddx58, Mmp9, Ccl2, Bcl3, Nfkbia, Nfkb2, Tnfaip3, Cxcl10, Nfkbie, Relb, Vcam1, Mcc	E
4 h	CHEMOKINE (C C MOTIF) LIGAND 2	1.24E-05	5	Ccl2, Ccl7, Nfkbia, Cxcl10, Vcam1	C; E
4 h	INTERLEUKIN 18 (INTERFERON GAMMA INDUCING FACTOR)	4.14E-05	4	Ccl2, Fas, Il1rn, Vcam1	E
4 h	INTERLEUKIN 1	5.62E-05	6	Mmp9, Ccl2, Nfkbia, Cxcl10, Il1rn, Vcam1	E
4 h	TNF RECEPTOR ASSOCIATED FACTOR	2.02E-04	5	Ddx58, Fas, Nfkbia, Nfkb2, Tnfaip3	E
4 h	RECEPTOR ACTIVATOR OF NUCLEAR FACTOR KAPPA B LIGAND	7.86E-04	5	Fas, Nfkbia, Tnfaip3, Nfkbie, Relb	
4 h	INTERLEUKIN 10	8.73E-04	4	Ddx58, Ccl2, Cxcl10, Il1rn	
4 h	CD40 LIGAND	9.38E-04	3	Fas, Nfkbia, Tnip1	
12 h	TUMOR NECROSIS FACTOR (TNF SUPERFAMILY, MEMBER 2)	8.98E-12	16	Mmp9, Ccl2, Cxcl1, Nfkbia, Ccl5, Cxcl10, Il1rn, Nfkbie, Relb, Vcam1, Saa3, Fas, Tnip1, Tnfaip3, Slc40a1, Serpinb2	E
12 h	NF KAPPA B	8.55E-11	18	Ddx58, Mmp9, Ccl2, Cxcl1, Nfkbia, Nfkb2, Stap2, Ccl5, Cxcl10, Nfkbie, Relb, Vcam1, Cd74, Mcc, Saa3, Bcl3, Tnfaip3, Capn6	E
12 h	CHEMOKINE (C C MOTIF) LIGAND 2	7.07E-07	7	Ccl2, Ccl7, Cxcl1, Nfkbia, Ccl5, Cxcl10, Vcam1	C; E
12 h	TOLL LIKE RECEPTOR	7.76E-05	7	Ddx58, Cxcl1, Stap2, Ccl5, Cxcl10, Saa3, Tnfaip3	
12 h	INTERLEUKIN 1	1.53E-04	7	Mmp9, Ccl2, Cxcl1, Nfkbia, Cxcl10, Il1rn, Vcam1	E
12 h	TNF RECEPTOR ASSOCIATED FACTOR	3.08E-04	6	Ddx58, Nfkbia, Nfkb2, Stap2, Fas, Tnfaip3	E
12 h	INTERLEUKIN 18 (INTERFERON GAMMA INDUCING FACTOR)	3.22E-04	4	Ccl2, Il1rn, Vcam1, Fas	E
12 h	MYELOID DIFFERENTIATION PRIMARY RESPONSE GENE (88)	9.46E-04	5	Ddx58, Cxcl1, Stap2, Ccl5, Cxcl10	E
12 h	RECEPTOR ACTIVATOR OF NUCLEAR FACTOR KAPPA B LIGAND	1.46E-03	6	Nfkbia, Ccl5, Nfkbie, Relb, Fas, Tnfaip3	
12 h	INTERLEUKIN 6 (INTERFERON, BETA 2)	2.81E-03	5	Ccl2, Cxcl1, Ccl5, Cxcl10, Cp	E
12 h	CD40 LIGAND	4.18E-03	3	Nfkbia, Fas, Tnip1	
12 h	INTERLEUKIN 10	5.99E-03	4	Ddx58, Ccl2, Cxcl10, Il1rn	
24 h	TUMOR NECROSIS FACTOR (TNF SUPERFAMILY, MEMBER 2)	3.25E-10	16	Mmp9, Cxcl1, Ccl5, Nfkbie, Vcam1, Saa3, Lbp, Slc40a1, Tnfaip3, Ccl2, Birc3, Nfkbia, Cxcl10, Relb, Tnip1, Serpinb2	E
24 h	NF KAPPA B	5.79E-10	19	Mmp9, Cxcl1, Ccl5, Nfkbie, Vcam1, Cd74, Saa3, Bcl3, Tnfaip3, Ccl2, Birc3, Nfkbia, Nfkb2, Stap2, Cxcl10, Relb, Mcc, Abcb1b, Capn6	E
24 h	CHEMOKINE (C C MOTIF) LIGAND 2	1.03E-08	9	Cxcr4, Cxcl1, Ccl5, Vcam1, Ccl7, Ccl2, Nfkbia, Cxcl10, Abcb1b	C; E
24 h	TOLL LIKE RECEPTOR	2.92E-04	7	Cxcl1, Ccl5, Saa3, Lbp, Tnfaip3, Stap2, Cxcl10	
24 h	INTERLEUKIN 1	3.15E-03	6	Mmp9, Cxcl1, Vcam1, Ccl2, Nfkbia, Cxcl10	E
24 h	MATRIX METALLOPROTEINASE	4.08E-03	6	Mmp9, Cxcr4, Ccl5, Enpp2, Postn, Adamts5	C
24 h	TNF RECEPTOR ASSOCIATED FACTOR	5.66E-03	5	Tnfaip3, Birc3, Nfkbia, Nfkb2, Stap2	E
24 h	INTERLEUKIN 6 (INTERFERON, BETA 2)	6.82E-03	5	Cxcl1, Ccl5, Ccl2, Cxcl10, Cp	E

Signal transduction pathway associations, which were enriched after 4 h (“induction of differentiation”/immediate response), and 12 h (very early differentiation) of treatment, and 24 h (early differentiation) treatment, are depicted. The effects of differentiation without or with TNF-α or with IGF1 compared with TNF-α treatment are shown. Pathway enrichment was based on cocitation with a p value cutoff of <0.01. Genes within significantly enriched pathways are listed. In addition, it is indicated in which SOTA cluster a pathway is enriched. Pathways highlighted in bold are retrieved in enrichment analyses of genes identified by principal component analysis which are shown in Table 1.

In contrast to the effect of myoblast differentiation, the effect of TNF-α treatment on gene expression, and thus pathway enrichment, was approximately constant over time (Table 3). However, slight time-specific enrichment of signal transduction pathway associations were evident as the number of signal transduction pathway associations peaks at 12 h after induction of differentiation. Signal transduction pathway association analysis of genes regulated by TNF-α during myoblast differentiation revealed that the following pathways enriched at 4 h, 12 h as well as at 24 h included tumor necrosis factor (TNF superfamily, member 2), NFkB, and chemokine (C C motif) ligand 2 (Table 3). Moreover, TNF-α treatment regulated matrix metalloproteinase signaling after 24 h of TNF-α and differentiation stimuli. Genes with a function in TNF-α or cytokine signaling were retrieved in SOTA cluster E (specifically induced by TNF). Genes upregulated by TNF-α after 24-h incubation had a function in the chemokine (CC motif) ligand 2 or matrix metalloproteinase pathway, which are both enriched in SOTA cluster C (early myotube genes down). Furthermore, the effect of IGF1 compared with TNF-α revealed enrichment of similar pathways as observed for the effect of TNF-α compared with the untreated control (Table 3). In summary, enrichment of several pathways was validated across methods (compare Tables 1 and 3). Inter method validated pathways were highlighted in bold (Tables 1 and 3). Pathways which did not match between results from principal component analysis and results from differential gene expression analysis resemble the consequence of different analyses approaches.

### TNF-α inversely regulated early differentiation-associated genes

TNF-α impaired myoblast differentiation; therefore, we aimed to identify differentiation-associated genes inversely regulated by TNF-α. We identified genes counteracted by TNF-α (Table 4) after 24-h treatment; this included several genes that were among the top 20 most upregulated genes during differentiation, such as Cpa1, Aspn, Adamts5, and Fibin. Most of the inversely regulated genes were upregulated during differentiation but downregulated because of TNF-α treatment (Table 4).

**Table 4:** TNF-α inversely regulated differentiation genes.

Gene Symbol	Gene Title	log2 ratio differentiation	log2 ratio TNF-α	Pathway association (differentiation)	Pathway association (TNF-α)	Literature background
Cpa1	carboxypeptidase A1	3.56	-1.57			M
Aspn	asporin	2.74	-1.80	Mothers against Dpp homolog, TGFβ		SkM
Adamts5	a disintegrin-like and metallopeptidase (reprolysin type) with thrombospondin type 1 motif, 5 (aggrecanase-2)	2.66	-1.28	TGFβ	Matrix metallo proteinase	SkMDiff
Fibin	fin bud initiation factor homolog (zebrafish)	2.65	-1.10			SkM
Trdn	triadin	2.45	-1.05	Ryanodine receptor		SkMDiff
Slc40a1	solute carrier family 40 (iron-regulated transporter), member 1	2.34	-1.85		Tumor necrosis factor (TNF superfamily member 2)	SkM
Capn6	calpain 6	2.20	-2.21		NF kappa B	SkMDiff
Nrk	Nik related kinase	2.06	-1.25			SkM
Cmbl	carboxymethylenebutenolidase-like (Pseudomonas)	2.04	-1.36			new
Aknad1	AKNA domain containing 1	2.03	-1.32			new
Parm1	prostate androgen-regulated mucin-like protein 1	2.00	-1.69			M
Itm2a	integral membrane protein 2A	1.99	-1.68			SkMDiff
Sepp1	selenoprotein P, plasma, 1	1.87	-1.06			new
Ndst4	N-deacetylase/N-sulfotransferase (heparin glucosaminyl) 4	1.73	-1.29			new
Tecrl	trans-2,3-enoyl-CoA reductase-like	1.66	-1.40			new
Cnr1	cannabinoid receptor 1 (brain)	1.64	-1.03			SkMDiff
Unc13c	unc-13 homolog C (C. elegans)	1.62	-1.71			new
Spats2l	spermatogenesis associated, serine-rich 2-like	1.51	-1.07			new
Mybpc1	myosin binding protein C, slow-type	1.34	-1.03			SkM
Lix1	limb expression 1 homolog (chicken)	1.16	-1.42			new
Csdc2	cold shock domain containing C2, RNA binding	1.15	-1.19			new
Fzd4	frizzled homolog 4 (Drosophila)	1.04	-1.26			SkMDiff
Serpinb2	serine (or cysteine) peptidase inhibitor, clade B, member 2	-2.28	1.74		Tumor necrosis factor (TNF superfamily member 2)	M

List of genes upregulated by differentiation but downregulated because of TNF-α treatment or vice versa. Log2 ratios indicate the order of magnitude of differential expression. Enriched signal transduction pathway associations in which the respective gene is involved during differentiation or TNF-α treatment are shown. The literature background indicates whether the gene has been published in skeletal muscle differentiation (SkMDiff), skeletal muscle (SkM), heart muscle, smooth muscle, or muscle progenitor cells (M), or whether it has not been described in muscle (new).

Only one gene, Serpinb2, was downregulated during myoblast differentiation but upregulated upon TNF-α stimulus (Table 4). Inversely, regulated genes were indicative of which pathways may be counteracted by TNF-α that lead to the observed phenotypic impairment of differentiation [27]. These genes included Aspn, Adamts5, Trdn, Slc40a1, Capn6, and Serpinb2, which are involved in the following enriched pathways: mothers against DPP homolog, TGF beta, matrix metalloproteinase, ryanodine receptor, tumor necrosis factor (TNF superfamily, member 2), NFkB, or TNF (compare Tables 3 and 4).

### Gene expression profiling results were validated at the mRNA and the protein level

**Fig 4:**
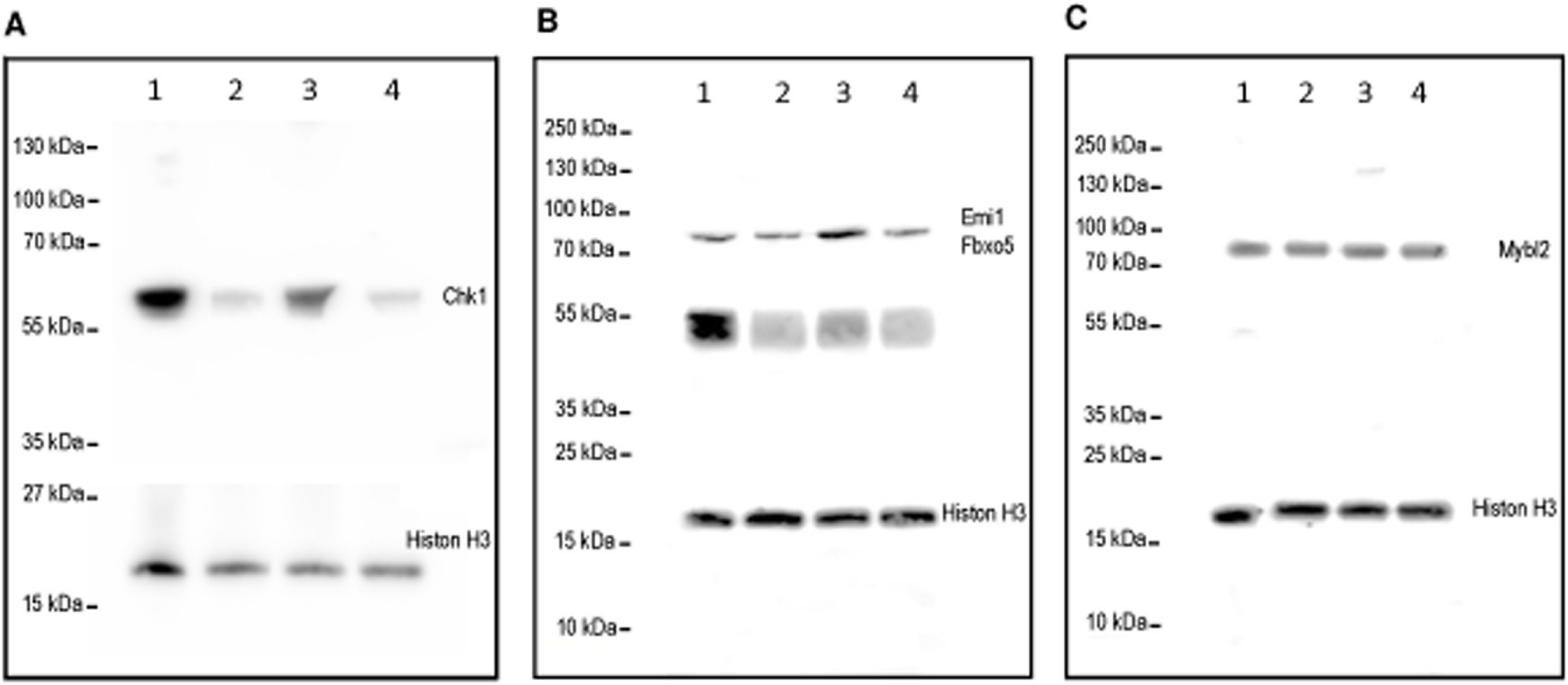
Western Blot analysis of differentially expressed genes. (A) Chk1, (B) Emi1/Fbxo5, (C) Mybl2 protein were detected by western blot analysis. Histone H3 served as the normalization control. Murine mouse muscle cells were cultured for 48 h in growth medium (lane 1), differentiation medium (lane 2), or differentiation medium supplemented with TNF-α (lane 3) or IGF1 (lane 4), respectively. (B) The specificity of the double band between 40 and 50 kDa was confirmed by peptide competition of the Emi/Fbxo5 antibody epitope (S4 Fig).

Gene expression profiling results were validated by RT-qPCR analysis (S3A–S3D Fig) as indicated by Pearson correlation coefficient values between 0.94 and 0.89 during differentiation and TNF-α treatment. When the same genes were measured for the effect of IGF1 compared with the untreated control, the correlation coefficient value was 0.53 because of small fold-changes below 1.5 with in part inverse algebraic sign. Multiple regression analysis for relative expression values of microarray and individual RT-qPCR analysis (S3E Fig) showed higher dynamics of RT-qPCR results compared with microarray data. Gene expression profiling results were validated at the protein level as depicted for the Serine/threonine-protein kinase Chk1 (Fig 4A) and F-box protein 5 (Fbxo5/Emi1) (Fig 4B). Despite the downregulation of Mybl2 mRNA during differentiation, there was no significant regulation of Mybl2 protein as confirmed by western blot analysis (Fig 4C). Selection criteria for genes which were analyzed by RT-qPCR or western blot were based on the integrative analysis of microRNA and mRNA expression data as described by Meyer et al. [28,29].

## Discussion

Gene expression kinetics of *in vitro* myoblast differentiation in the presence of IGF1 or inflammatory levels of TNF-α have not yet been described in detail. Based on microarray data of PMI28 myoblasts, the current study elucidated gene expression kinetics and its networks immediately after induction of differentiation (4 h), during very early (12 h), and early (24 h) differentiation as well as late (72 h) differentiation. Results from the current study indicated significant effects of TNF-α and subtle changes in gene regulation because of IGF1 treatment. Thus, the discussion section focuses on the effects observed for TNF-α treatment on gene expression of differentiating myoblasts.

### Immediate response to differentiation as well as TNF-α and coregulation of gene sets

The current study detected significant co-regulation of gene sets as well as an immediate and specific response to TNF-α, which interfered with gene expression regulation during normal differentiation. In summary, the vast majority of genes differentially regulated in myoblast differentiation and response to TNF-α or IGF1 were upregulated during early or late differentiation. Our findings relate to Henningsen et al. [2] who reported that a higher proportion of muscle-released proteins exhibited an increased level of secretion compared with the proteins with a decreased secretion profile during the course of C2C12 differentiation. Moreover, we found that genes with similar relative expression profiles were enriched for genes with similar biological implications indicating significant co-regulation of functionally related gene sets.

Genes upregulated during very early and or late myoblast differentiation were associated with muscle cell differentiation, muscle structure development, or muscle contraction, which is in agreement with the observed phenotypic differentiation [27] including withdrawal from the cell cycle, myoblast fusion, and formation of multinucleated myotubes. In harmony with this, we identified accumulation of coexpressed genes belonging to pathways which are upregulated during differentiation or which are positive regulators of differentiation such as cyclin G1 [30], semaphorin [31–34,2], ryanodine receptor [35,36], calcineurin (protein level: [37], activity level: [38]), and doublecortin like kinase.

We propose that one of the inhibitory effects of TNF-α on myoblast fusion could be associated with NF-kappaB activation and ryanodine receptor regulation. This assumption is based on a study by Valdes et al. [39], which suggested that NF-kappaB activation in skeletal muscle cells is linked to membrane depolarization and depends on sequential activation of calcium release mediated by the ryanodine and by IP(3) receptors [39]. Moreover, RyR1 alters the expression pattern of several proteins involved in calcium homeostasis [40], which regulates calcineurin amongst others. Calcineurin may have therapeutic potential, as Stupka et al. [41] demonstrated that calcineurin is essential for skeletal muscle regeneration in wild type mice or in young mdx mice in which calcineurin stimulation can ameliorate the dystrophic pathology [41]. Moreover, after 24 h of differentiation, pathways including the doublecortin like kinase pathway were enriched. Doublecortin like kinase encodes a microtubule-binding protein. To date, the doublecortin like kinase pathway has not been discussed in the context of myoblast differentiation or response of differentiating myoblasts to TNF-α. We speculate that doublecortin like kinase may play a role in myoblast migration or guidance as it has been known that doublecortin like kinase is associated with interneuron migration [42] and axon guidance [43].

#### Genes downregulated during early myotube formation

Clustered coexpression of genes, which were down-regulated during early differentiation, were enriched for genes involved in distinct signaling associations. The following signaling pathways have been described in muscle cell differentiation: mothers against DPP homolog [44], matrix metalloproteinase [45–48], peroxisome proliferator activated receptor delta [49], very low density lipoprotein receptor [50], dual specificity phosphatase [51], fibroblast growth factor [52–55], TGF beta [56–60], and hypoxia inducible factor 1 alpha subunit [61]. Moreover, LDL receptor-related protein (LRP-1) and decorin were modulators of the TGF-β-dependent signaling pathway [62]. It was reported that the TGF-beta intracellular effector Smad3 mediates the inhibition of myogenic differentiation by repressing the activity of the myogenic transcription factors [63,64]. For the dual specificity phosphatase pathway, it has been shown that estrogen-related receptor alpha regulated the transient induction of MAP kinase phosphatase-1/dual specificity phosphatase at the onset of myogenesis, which mediated ERK dephosphorylation and promoted myotube formation [51]. In contrast, our data revealed downregulation of Dusp4, Dusp5, and Dusp9 during myoblast differentiation, which has not yet been discussed in the context of myocytes.

#### Genes downregulated during late myotube formation

Moreover, signal transduction pathway associations of coregulated clustering genes, which decreased in expression during later differentiation, included nuclear factor (erythroid derived 2)-like two (Nrf2), tumor protein p53, breast cancer 1 early onset (Brca1) as well as the cell division cycle. Consistent with a role of the Nrf2 pathway in myogenic differentiation, it has been reported that Nrf2 protein expression increased during myogenesis and regulated muscle differentiation [65]. Nrf2 promoted muscle regeneration and protected against TWEAK-mediated muscle wasting [66]. However, our data shows down-regulation of Nrf2 signal pathway associations. Furthermore, p53 signal transduction pathway associations were in agreement with the finding that p53 activation was measurable during myoblast differentiation and that p53 had a specific role in this process [67–69]. Moreover, Brca1 was involved in cell differentiation, and it has been shown to be upregulated during C2C12 myoblast differentiation [70]. However, our data is contradictory to the findings of Kubista et al. [70] as we detected downregulation of Brca1. In addition, the gene ontology term, cell cycle, was significantly enriched in genes downregulated during later differentiation, which is represented by serin/threonine-protein kinase (Chk1) gene expression for example. Chk1 was associated with several enriched signal transduction pathways, including breast cancer 1 early onset and tumor protein 53. Chk1 activity was associated with regulation of cell cycle and differentiation [71] in other cell types. The known functions of Chk1 are discussed in paragraph “Gene expression profiling results were validated at the mRNA and protein level”.

#### TNF-induced and suppressed genes during late myotube formation

TNFα-induced genes downregulated during late myoblast differentiation were of special interest as they were modulated by TNF-α, and at the same time essential for skeletal muscle cell differentiation. These genes may point to possible therapeutic strategies to ameliorate the inhibitory effect of TNF-α. In harmony with this assumption, we found gene ontology biological process terms enriched, which are associated with regulation of cell proliferation, differentiation, migration, and motility. Interleukin 1 receptor antagonist amongst others was upregulated by TNF-α but downregulated during differentiation, which was in harmony with a known positive effect of IL-1 on myogenic differentiation [72]. Moreover, Cdk6 expression regulation was significantly associated with signal transduction pathway cyclin dependent kinase inhibitor 2. In agreement with this, it has been known that myoblast cell cycle exit and differentiation are mediated in part by down-regulation of cyclin D1 and associated cyclin-dependent kinase (Cdk) activity [73]. Consistent with a role for Cdk4/Cdk6 activity as a regulator of myogenic differentiation, Saab et al. [73] observed that Cdk4/Cdk6 inhibition promoted morphologic changes in myoblasts and enhanced the expression of muscle-specific proteins [73].

#### TNF-specific induced genes

Genes specifically induced by TNF-α were involved in the immune response and were associated with signal transduction pathway associations such as NF kappa B, TNF-α signaling, chemokine (C C motive) ligand 2, toll like receptor, IL-1, IL-6, and IL-18. More importantly, these pathways have been associated with cell proliferation and differentiation. Inflammatory cytokines such as TNF-α have been known to inhibit myogenic differentiation, in part through sustained NF-kappaB activity [9]. Activated NF-kappaB interfered with the expression of muscle proteins in differentiating myoblasts [9] by inducing loss of MyoD mRNA [74] or interference with the function of MyoD [75]. Moreover, NF-kappaB activates cyclin D1 expression at the transcriptional level, which inhibits myogenesis [76] and regulated cyclin D1 protein D1 stability [77]. In addition, our data revealed that TNF-α exposure increased gene expressions associated with the IL-1 pathway in differentiating myotubes. Grabiec et al. [72] reported that interleukin-1beta stimulated early myogenesis of mouse C2C12 myoblasts, and concluded that IL-1beta was associated with the impact on myogenic regulatory factors [72]. On the other hand, IL-1beta induced Id2 gene expression in vascular smooth muscle cells [78], which could point to an inhibitory effect of IL-1beta in skeletal muscle cells. Furthermore, TNF-α specifically induced expressions were enriched for genes associated with the IL-6 and IL-18 pathways. It has been reported that TNF-α exposure increased IL-6 in skeletal myoblasts [79,80]. IL-6 increased myogenic differentiation [81] and the mRNA expression of myocyte enhancer factor 2D [82], while IL-6 has been known to stimulate myoblast proliferation [83,84]. It has been shown that IL-18 stimulated airway smooth muscle cell proliferation [85] and activated NF-kappaB amongst others [86]. Our data suggested that IL-1, IL-6, and IL-18 pathway associations could be mediators of the inhibitory effect of TNF-α on skeletal muscle differentiation, or may have implications in compensating for anti-myogenic effect of the pathological concentrations of TNF-α levels.

In summary, we confirmed known gene regulations and identified new genes, which have not yet been described, to play a role in mediating the response to TNF-α in skeletal myoblast differentiation. Moreover, we provided kinetic gene expression data of the very early and early differentiation response, which facilitated the understanding of the regulatory networks, leading to impaired myoblast fusion upon pathological concentrations of TNF-α. Coregulated gene sets were enriched for pathways, which have been described in the context of myoblast differentiation. However, our data showed new avenues in the complexity of gene expression kinetics and networks, and pointed to findings contradicting the current literature on first sight. Moreover, we have identified TNF-α-regulated genes in skeletal muscle cell differentiation, which have not been implicated in this process before. An increased understanding of gene expression regulation during skeletal muscle cell differentiation may provide new approaches for the development of strategies to counteract impaired muscle regeneration or muscle wasting.

### Specific signaling pathway regulation during myoblast differentiation and TNF-α response

Differential gene expression kinetics revealed dynamic, time-specific change of gene regulation as well as genes constantly downregulated immediately subsequent induction and during the course of differentiation, including mothers against dpp homolog signaling or semaphorin signaling associated genes. Our data confirmed the importance of regulating mothers against dpp homolog or Smad protein signaling in myoblast differentiation. Moreover, semaphorins have been linked to muscle regeneration [34]. However, the upregulated isoforms of semaphorins identified within the current study, namely Sema6a, Sema6d, Sema5a, and Sema3c, have not yet been described in myoblast differentiation. The majority of differentially regulated genes were enriched during signal transduction pathway associations in a time-dependent manner. Induction of myoblast differentiation can be characterized as being regulated by genes involved in mothers against DPP homolog and TGFbeta signaling. With Smad proteins being downstream mediators of TGFbeta signaling, our data emphasize the role of TGFbeta and downstream Smad signaling in modulating myoblast differentiation and myotube formation (compare [56]). After 12 h, differentiation genes involved in signaling pathways such as mothers against DPP homolog, notch, semaphorin, cadherin, TGF beta, and fibroblast growth factor were enriched. Thus, we can conclude that TGFbeta signaling was a major regulatory pathways during the first hours (4 h–12 h) of differentiation. After 24 h of differentiation we identified enrichment of pathway associations including notch signaling, cyclin, cyclin dependent kinase, and cycline dependent kinase inhibitor amongst others. Thus, notch signaling can be attributed to the differentiation phase from 12 h to 24 h of differentiation, whereas cell cycle regulation is the predominant theme after 24 h differentiation.

TNF-α treatment specifically upregulates several genes immediately after induction (4 h), which remain upregulated after 12 h as well as 24 h incubation in differentiation medium, and which are related to TNF, NFkB, chemokine ligand, and interleukin pathways in agreement with immune responsive reactions of muscle cells. Moreover, after 24 h, TNF-α treatment regulated genes associated with matrix metalloproteinase which may indicate that TNF-α excerts part of its pro-proliferative functions through modulating the MMP pathway and thus myoblast migration.

### TNF-α inversely regulated early differentiation-associated genes

It is of particular interest to identify genes inversely regulated in myogenic differentiation compared with TNF-α treated differentiating myoblasts. We identified eleven TNFα-regulated genes which have been previously described in the context of skeletal muscle, including Aspn [87], Adamts5 [88], Fibin [89], Triadin [90], Slc40a1 [91], Capn6 [92], Nrk [93], Itm2a [94], Cnr1 [95], Mybpc1 [96], and Fzd4 [97]. Of these eleven genes, only six have previously been described in skeletal muscle differentiation or regeneration: Adamts 5 [88,98], Triadin [90,99], Cnr1 [95], Itm2a [94], Fzd4 [97], and Capn6 [100].

**Fig 5:**
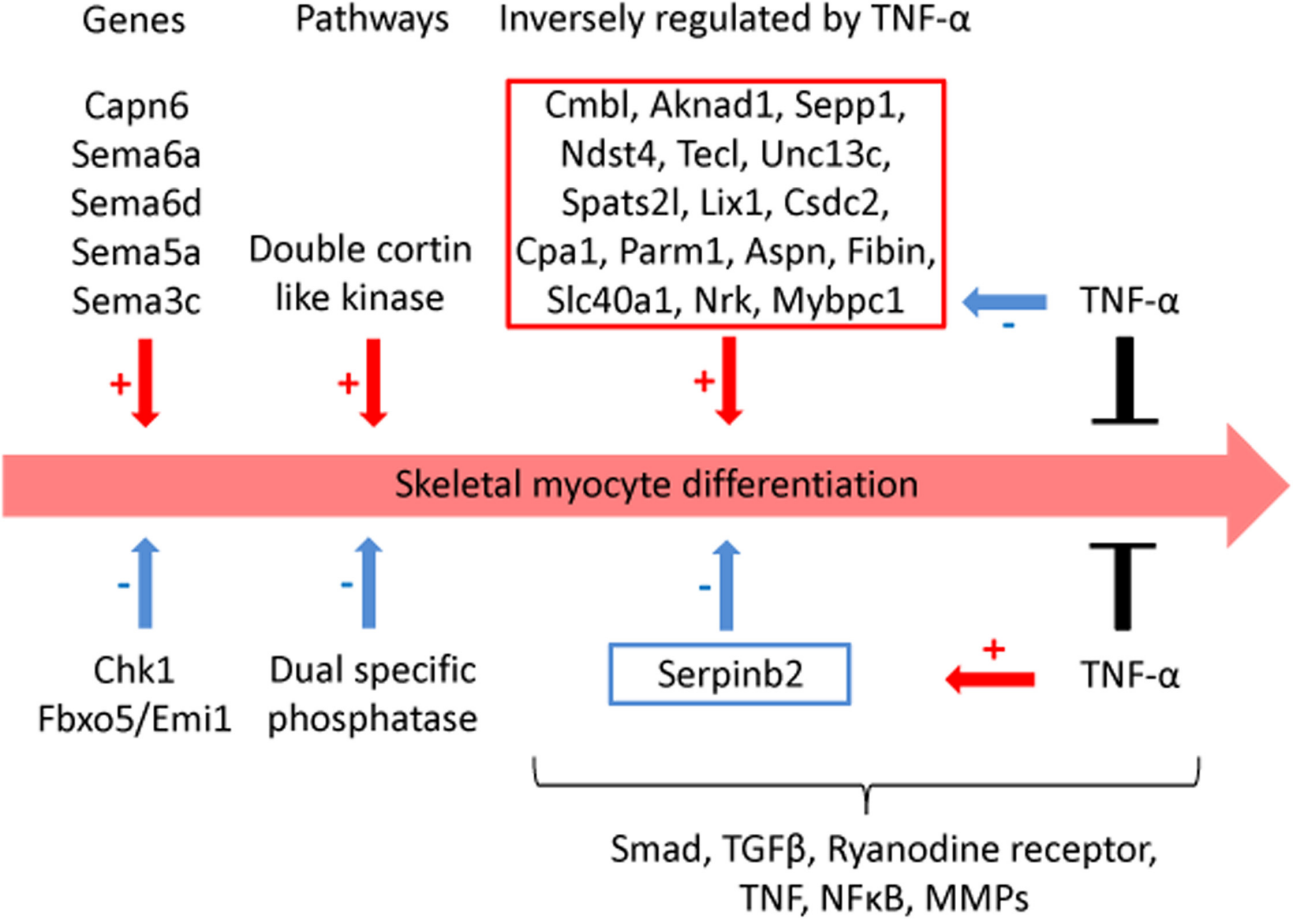
Novel genes and pathways in skeletal myocyte differentiation and TNF-α response. We identified genes and pathway associations, which have not been described before in skeletal myocyte differentiation or have been reported to have a different regulation than the one observed in the current study. A plus indicates upregulation during differentiation and a minus indicates downregulation. Moreover, we show genes which are inversely regulated by TNF-α, but have not been defined before, to be regulated in skeletal myocyte differentiation and response to TNF-α.

However, it has not yet been reported that Adamts5 was one of the 20 most up-regulated genes during differentiation and that its expression was negatively regulated by TNF-α during myogenic differentiation. Furthermore, the described expression regulation or known functions of triadin, Adamts5, Cnr1, Itm2a, and Fzd4 in skeletal muscle differentiation underline the significance of our findings that TNF-α deregulated these genes during myogenic differentiation and reduced fusion capacity of myoblasts. However, our findings regarding Capn6 expression regulation were contradictory to a study by Tonami et al. [100] reporting that Capn6 was a suppressor of skeletal muscle differentiation, and a study by Liu et al. [101] indicating that Capn6 promoted cancer cell proliferation and was positively regulated by the PI3K-Akt signaling pathway. On the other hand, it has been shown that Capn6 expression was suppressed by serum in fibroblast cell culture [100], which would be in harmony with the upregulation of Capn6 upon serum deprivation observed in the current study. Remarkably, this effect was reversed by TNF-α treatment. Other members of the calpain family have been discussed in myocyte differentiation, namely Capn1, which has been reported to play an important role for satellite cell myogenesis [102], and Capn2 / m-calpain, which had been shown to play a role in the control of muscle precursor cell differentiation [103]. Therefore, we hypothesize that Capn6 could be a myofusion marker which may participate in promoting *in vitro* differentiation of skeletal myoblasts through an unknown physiological mechanism. We identified inversely regulated genes described in the context of skeletal muscle but not in the context of myoblast differentiation or TNF-α response such as Aspn, Fibin, Slc40a1, Nrk, and Mybpc1 (Fig 5). To date, Aspn had been described in the context of congenital muscular corticollis [87], cardiac remodeling [104], or the transition from a hyperplasic myotube-producing phenotype to a hypertrophic growth phenotype in fish [105]. The current study is the first identifying Aspn expression regulation in the context of skeletal myogenic differentiation and its response to TNF-α as well as enrichment in TGFbeta signaling pathway associations in skeletal muscle. Similarly to Aspn, we detected fibin among the top 20 most upregulated genes during myoblast differentiation. It is reported that fibin is expressed in skeletal muscle [89] amongst other tissues. We provided indications for a role of fibin in the regulation of myogenic differentiation and its response to TNF-α. Slc40a1, which encoded ferroportin [106], was hypothesized to influence skeletal muscle iron content [91]. We found that Slca40a1 was associated with the tumor necrosis factor pathway (Table 3), but the current study is the first describing a role of Slca40a1 in myogenic differentiation.

We observed upregulation of Nrk/Nik related kinase during differentiation and downregulation by TNF-α treatment. Nrk has been known to be expressed in skeletal muscle during mouse embryogenesis [107,93] and may be involved in the regulation of actin cytoskeletal organization in skeletal muscle cells through cofilin phosphorylation and actin polymerization [93]. However, the current study is the first describing Nrk/Nik regulation during *in vitro* skeletal myoblast differentiation and TNF-α response. Myosin binding protein C (MyBP-C) is expressed in striated muscles where it modulates actomyosin cross-bridges [96] and acts as an adaptor to connect myosin and muscle-type creatine kinase for efficient energy metabolism and homoeostasis [108]. Mutations of sMyBP-C have been causally linked to the development of distal arthrogryposis-1, a severe skeletal muscle disorder [96,109], and lethal congenital contracture syndrome type 4 [109]. We describe Mybpc1 upregulation in the context of *in vitro* myoblast differentiation, which is counteracted by TNF-α.

Importantly, we identified genes inversely regulated during skeletal myocyte differentiation compared with differentiation under TNF-α stimulus, but have not yet been described in the context of skeletal muscle or TNF-α effect on muscle cells. Some differentiation and TNF-α-regulated genes have been described in smooth or heart muscle, or muscle progenitor cells, but not in skeletal muscle, including Cpa1 [110], Parm1 [111], and Serpinb2 [112] (Fig 5). Parm1 expression was detected in the muscle progenitor cells of the somites [111]. Thus, the role of Parm1 in muscle differentiation and TNF-α response still needs to be unraveled. Serpinb2 was >4-fold downregulated during differentiation, but upregulated during TNF-α treatment. There has not yet been evidence for the observed effect in skeletal myoblast differentiation. However, in smooth muscle, Jang et al. [112] have reported that plasminogen activator inhibitor-2 protein levels were upregulated by TNF-α. It has been reported that PAI-2 (Serpinb2) is upregulated during cell cycle progression in myoepithelial cells [113]. Moreover PAI reduced the capacity of endothelial cells to lyse fibrin [114] and rPAI-2-expressing sarcoma cells showed inhibited invasion characteristics [115]. We speculate that downregulation of Serbinb2 in myoblast differentiation may facilitate cell migration during the early phase of differentiation.

The current study reveals nine genes regulated by differentiation and TNF-α, which have not yet been described in muscle cells. We describe for the first time a significant specific regulation of the following genes in myoblast differentiation: Aknad1, Sepp1, Ndst4, Tecrl, Cmbl, Unc13c, Spats2l, Lix1, and Csdc2 (Fig 5). On the basis of our expression data, we postulate a biological implication of these genes in myoblast differentiation and responsiveness to TNF-α. Comparatively little is known regarding the function of Aknad1 and Tecrl. Sepp1 is known to be involved in selenium distribution to tissues throughout the body [116]. Ndst4 is involved in N-sulfation of heparin sulfate [117] chains and is downregulated in carcinoma [118], indicating an anti-proliferative or pro-differentiative role. Moreover, a role of Cmbl has not yet been explicitly described in the muscular context. The physiological implications during skeletal muscle cell differentiation and its response to TNF-α remain elusive, but are likely to be of biological significance as Cmbl is one of the top 20 most regulated genes during myoblast differentiation within the current study. We identified significant expression regulation for Unc13c/Munc13-3 in myoblast differentiation. However, Munc13 has been described to be almost exclusively expressed in the cerebellum, which is a presynaptic protein [119] critical in regulating neurotransmitter release and synaptic plasticity [120]. In Munc13-deficient mice, the distribution, number, size, and shape of synapses, as well as the number of motor neurons they originate from and the maturation state of muscle cells, are profoundly altered [121]. The function of Spats2l in skeletal muscle differentiation and response to TNF-α has not been described earlier. Himes et al. [122] suggest that SPATS2L may be an important regulator of β(2)-adrenergic receptor downregulation. The function of Lix1 expression regulation in skeletal muscle cell differentiation remains elusive. It has been suggested that Lix1 plays a role in radial growth of motor axons observed in feline spinal muscular atrophy [123]. We identified inverse regulation of Csdc2 expression during differentiation and TNF-α treatment relative to control cells. However, Csdc2 has previously been described in the context of decidualization [124], but not skeletal muscle.

TNF-α dysregulated genes belong to signaling pathways enriched during myoblast differentiation, such as Smad, TGFβ, and ryanodine receptor, or TNF-α treatment such as TGFβ, matrix metalloproteinase, NFκB, and TNF. Dysregulations of these pathways have been known to be associated with muscular diseases [125–128,3].

### Gene expression profiling results were validated on the mRNA and the protein level

The expression of selected genes such as the Serine/threonine-protein kinase Chk1 [checkpoint kinase 1 homolog (*Schizosaccharomyces pombe*)] and F-box protein 5 (Fbxo5/Emi1) were validated on the protein level by western blot analysis. Both, Chk1, and Fbxo5 were downregulated during differentiation and in the IGF1 treatment group. However, when differentiation was induced in the presence of inflammatory levels of TNF-α Chk1 and Fbxo5/Emi1 were upregulated. The observed regulation of Chk1 on the protein level was in harmony with its known function as being part of a “G2 restriction point” that prevented premature cell cycle exit in cells programmed for terminal differentiation [71]. Chk1 functioned as a mitogen-dependent protein kinase that prevented premature differentiation of trophobast stem cells by suppressing expression of p21 and p57, but not p27, the CDK inhibitor that regulated mitotic cell cycles [71]. In the current study, we described for the first time Chk1 regulation during myoblast differentiation and its deregulation because of TNF-α treatment on the mRNA and protein level. Chk1 had been described in the context of estivating frogs, and it has been postulated that Chk1, amongst others, may contribute to preserving muscle function during metabolic depression and immobility [129]. In addition, we describe for the first time a specific regulation of Fbxo5/Emi1, both at the mRNA and the protein level, during myoblast differentiation and TNF-α treatment. Emi1 has been reported to play a role in somitogenesis [130]. Moreover, Emi1 functioned to promote cyclin A accumulation [131], and is known as a key cell-cycle regulator [132,131,133]. The described functions of Fbxo5/Emi1 were in harmony with the exit of the cell cycle of proliferating myoblasts to differentiate into myotubes. The inhibitory effect of TNF-α on myotube formation was resembled by diminished downregulation of Emi1/Fbxo5. Furthermore, we analyzed the protein level of transcription factor. In contrary to the upregulation of Mybl2 mRNA during proliferation and TNF-α treatment compared with differentiation control, there was no significant regulation of the Mybl2 protein. The latter may be because of protein stability and thus extended half-life of Mybl2 protein, which may mask the downregulation. Moreover, it was shown that Mybl2 was regulated post-transcriptionally [134] and phosphorylation was necessary to activate Mybl2 [135]. Further experiments need to analyze Mybl2 activity to possibly identify correlation with mRNA expression levels. Henningsen et al. [2] found little correlation between mRNA and protein levels for proteins secreted during myoblast differentiation. The latter indicates pronounced regulation by posttranscriptional mechanisms, such as functionality of miRNAs.

### New genes in TNF-α response during myoblast differentiation

Several of the differentially expressed genes identified in the current study were new in the context of impaired myoblast differentiation because of TNF-α exposure (compare S2B Table with S5 Table). However, a subset of genes has been confirmed in studies by Bhatnagar et al. [136] (S5A Table) or a study investigating TNF-like weak inducer of apoptosis (TWEAK) treatment by Panguluri et al. [137] (S5B Table). TWEAK mediated skeletal muscle wasting [66]. The common subset of genes between the different studies confirmed the validity of identified genes. In addition, the cross-study validated gene subset pointed to the most prominent genes, which were stably regulated across different murine skeletal myoblast cell lines and different treatment conditions. Bhatnagar et al. [136] analyzed C2C12 myoblasts treated with 10 ng/mL TNF-α, whereas the current study applied 5 ng/mL TNF-α. Genes differentially expressed in the current study as well as by Bhatnagar et al. [136] or Panguluri et al. [137] were enriched in the expression cluster (cluster B) containing genes slightly upregulated during early myotubes and highly upregulated during late myotubes (72 h) but downregulated by TNF-α or in expression cluster E which bore genes specifically induced by TNF-α. Thus, a gene subset can be confirmed across independent studies including genes which were i) involved in myotube differentiation but counteracted by TNF-α treatment or ii) TNFα-induced genes. Of note is that there was a common subset of genes regulated by TWEAK after 96 h of differentiation in C2C12 myotubes according to Panguluri et al. [137] and TNF-α treated PMI28 myotubes in the current study (S5B Table). Thus, TNF-α and TWEAK regulated in part common gene sets (S5C Table). Genes identified across different murine skeletal myoblast cell lines and different TNF-α treatment conditions as well as TWEAK treatment included Nfkbia, Nfkb2, Mmp9, Mef2c, Gpx, and Pgam2. Thus, several genes have not yet been described to play a role in the response to TNF-α in myoblast differentiation (S2B Table), while a small subset of genes had been confirmed by others (S5 Table).

## Conclusions

The understanding of the gene expression regulation during skeletal myoblast differentiation and how this is impacted by TNF-α and IGF1 is of significant clinical and therapeutic importance. Results of the current study facilitate the understanding of the regulatory networks leading to impaired myoblast fusion upon pathological concentrations of TNF-α. We confirmed genes and pathways prominent in myogenic differentiation or TNF-α signaling, and identified novel genes and pathways (Fig 5). Several differentiation-relevant genes were inversely regulated by TNF-α treatment. Moreover, our data revealed novel kinetic expression dynamics of genes and pathways during differentiation and TNF-α treatment. Moreover, TNF-α and IGF1 treatment could be characterized by a subset of indicative expression markers, of which some are robust inter-study retrieved markers. Results of the current study may point to possible candidates for new strategies to counteract impaired muscle regeneration in inflammatory myopathies, muscular dystrophies, or cancer cachexia. However, further research at the protein level is required.

## Supporting Information

S1 Fig Hierarchical clustering and heatmaps of gene expressions. After 24 h of treatment, the largest distance between groups appeared between myoblasts and myotubes, as well as myotubes exposed to TNF-α or IGF1.
(TIF)

S2 Fig Gene clusters which bear the minority of genes. The self-organizing tree algorithm clusters were given for genes that separated in (A) cluster G, containing five genes, (B) cluster H, including ten genes, and (C) cluster I, comprising 41 genes.
(TIF)

S3 Fig Expression analysis by qPCR validated gene profiling results. Relative fold-changes of Affymetrix gene expression profiling results and individual qPCR analysis results after 12 h of treatment are depicted for the effect of (A) differentiation, (B) TNF-α treatment, (C) IGF1 treatment, and (D) IGF1 treatment relative to TNF-α treatment. In each graph, the Pearson correlation coefficient R and the corresponding p values of microarray and qPCR results are shown. (E) Multiple regression analysis for relative expression values of microarray and qPCR analysis.
(TIF)

S4 Fig Peptide neutralization assay for Emi1. Western blot of Emi1/Fbxo5. (A) Emi 1 protein was detected at 46 kDa. However, we detected two unexpected bands at approximately 80 kDa and 110 kDa. (B) The Emi1-antibody was incubated with a 5-fold molar excess of Emi1 epitope rather than antibody. Specificity of the band at 46 kDa was confirmed as the band disappeared in contrary to the nonspecific bands at 80 kDa and 110 kDa.
(TIF)

S1 Table Gene lists of principal components. Gene lists of principal components of gene expression profiling data after 0 h,4 h, 12 h, and 24 h of differentiation and treatment with TNF-α, IGF1, or control.
(XLSX)

S2 Table Differential gene expression in myoblast differentiation and response to TNF-α or IGF1 treatment. Differential gene expression of nonambiguous genes derived from microarray analysis were listed for samples with 4 h (“induction of differentiation”/immediate response), 12 h (very early differentiation), and 24 h (early differentiation) of treatment. Corresponding log2 ratios are shown. (A) Differentiation, (B) TNF-α treatment, and (C) IGF1 compared with TNF-α treatment.
(XLSX)

S3 Table Genes which cluster by self-organizing tree algorithm analysis. Lists of genes are shown for (A) cluster A: upregulated during very early differentiation, (B) cluster B: genes upregulated during later differentiation, (C) cluster C: genes downregulated during very early differentiation, (D) cluster D: TNFα-induced and suppressed in late myotubes, (E) cluster E: genes specifically induced by TNF-α, (F) cluster F: late myotubes genes downregulated, (G) cluster G: upregulated by TNF-α and downregulated in late myotubes, (H) cluster H: upregulated in early myotubes, but downregulated in late myotubes, (I) cluster I.
(XLSX)

S4 Table Gene ontology and pathway enrichment of genes clustering by self-organizing tree algorithm analysis. Gene ontology biological process and signal transduction pathway associations are given for genes separated in clusters (A) cluster A: upregulated during very early differentiation, (B) cluster B, genes upregulated during later differentiation, (C) cluster C: genes downregulated during very early differentiation, (D) cluster D: TNFα-induced and suppressed in late myotubes, (E) cluster E: genes specifically induced by TNF-α, (F) cluster F: late myotubes genes downregulated, (G) cluster G: upregulated by TNF-α and downregulated in late myotubes, (H) cluster H: upregulated in early myotubes, but downregulated in late myotubes, (I) cluster I.
(XLSX)

S5 Table Comparison of the effect of TNF-α with previous studies. Genes differentially expressed in skeletal muscle cells in the current study and previous profiling studies are listed. (A) Genes differentially expressed in a study by Bhatnagar et al. [136] (C2C12, 18 h, 10 ng/mL TNF-α) as well as in the current study (PMI28, 4 h, 12 h, 24 h, 72 h, 5 ng/mL TNF-α) and the SOTA cluster in which the respective gene was retrieved (B) Regulated genes identified in a study by Panguluri et al. [137] (C2C12, 96 h, 10 ng/mL TWEAK) and in the current study (PMI28, 4 h, 12 h, 24 h, 72 h, and 5 ng/mL TNF-α) as well as the SOTA cluster in which the respective gene was retrieved. (C) Genes published by Bhatnagar et al. [136] and Panguluri et al. [137]. Bold genes were detected by Bhatnagar et al. (2010) [136], Panguluri et al. (2009) [137] as well as in the current study.
(XLSX)
