# Supplementary figures and images for: Tumor Necrosis Factor Alpha and Insulin-Like Growth Factor 1 Induced Modifications of the Gene Expression Kinetics of Differentiating Skeletal Muscle Cells
Source: PLoS One. 2015 Oct 8;10(10):e0139520. doi: 10.1371/journal.pone.0139520 (PMC4598026; doi:10.1371/journal.pone.0139520)

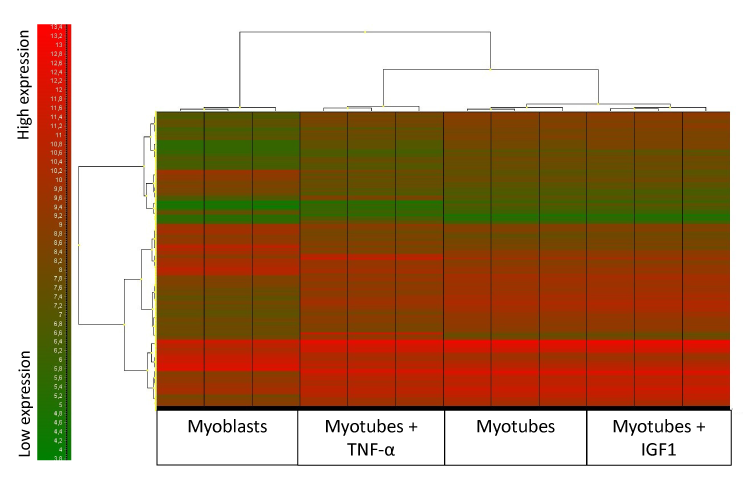

Supplement: S1 Fig — After 24 h of treatment, the largest distance between groups appeared between myoblasts and myotubes, as well as myotubes exposed to TNF-α or IGF1. (TIF) [file pone.0139520.s001.tif]

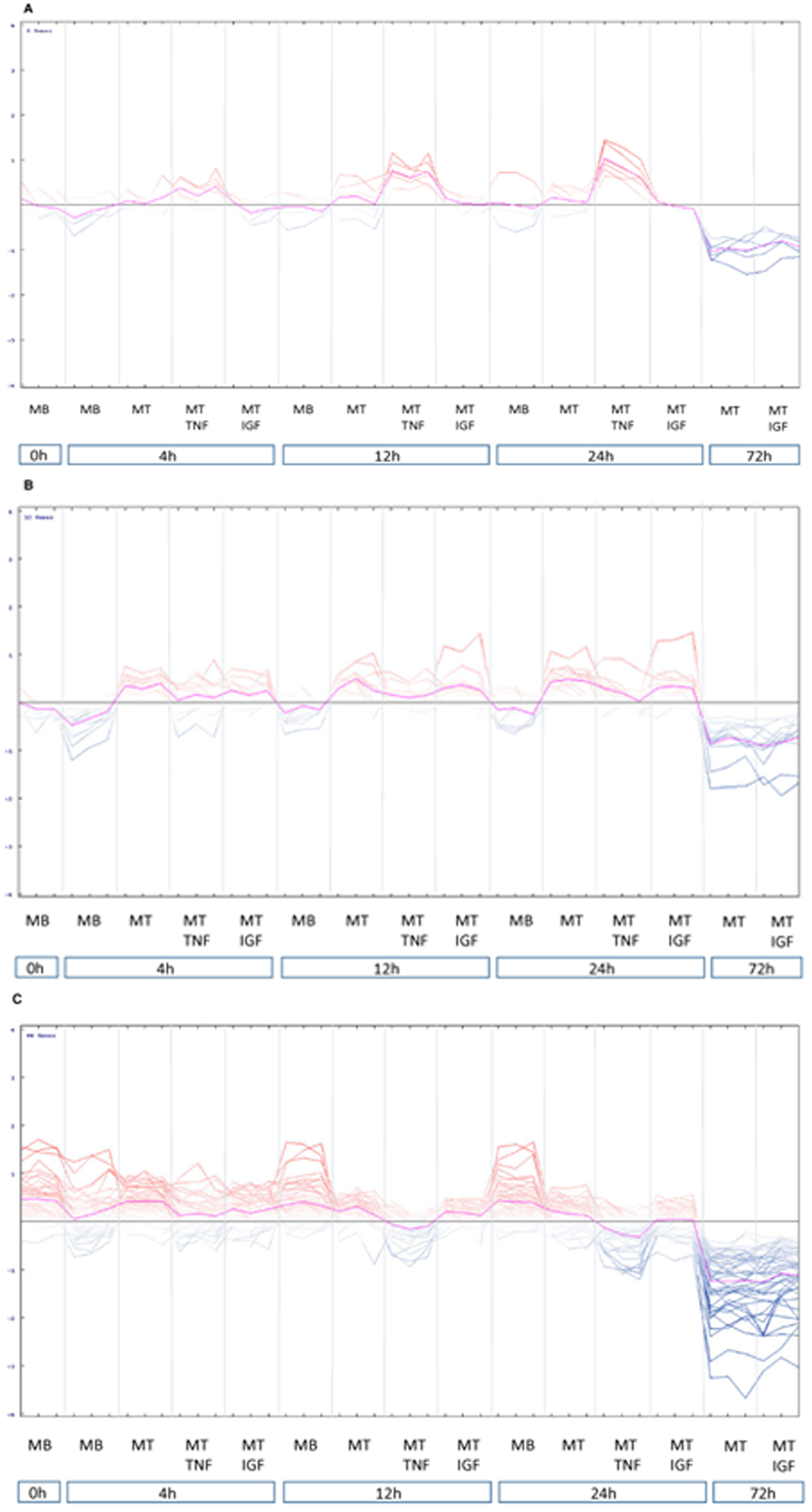

Supplement: S2 Fig — The self-organizing tree algorithm clusters were given for genes that separated in (A) cluster G, containing five genes, (B) cluster H, including ten genes, and (C) cluster I, comprising 41 genes. (TIF) [file pone.0139520.s002.tif]

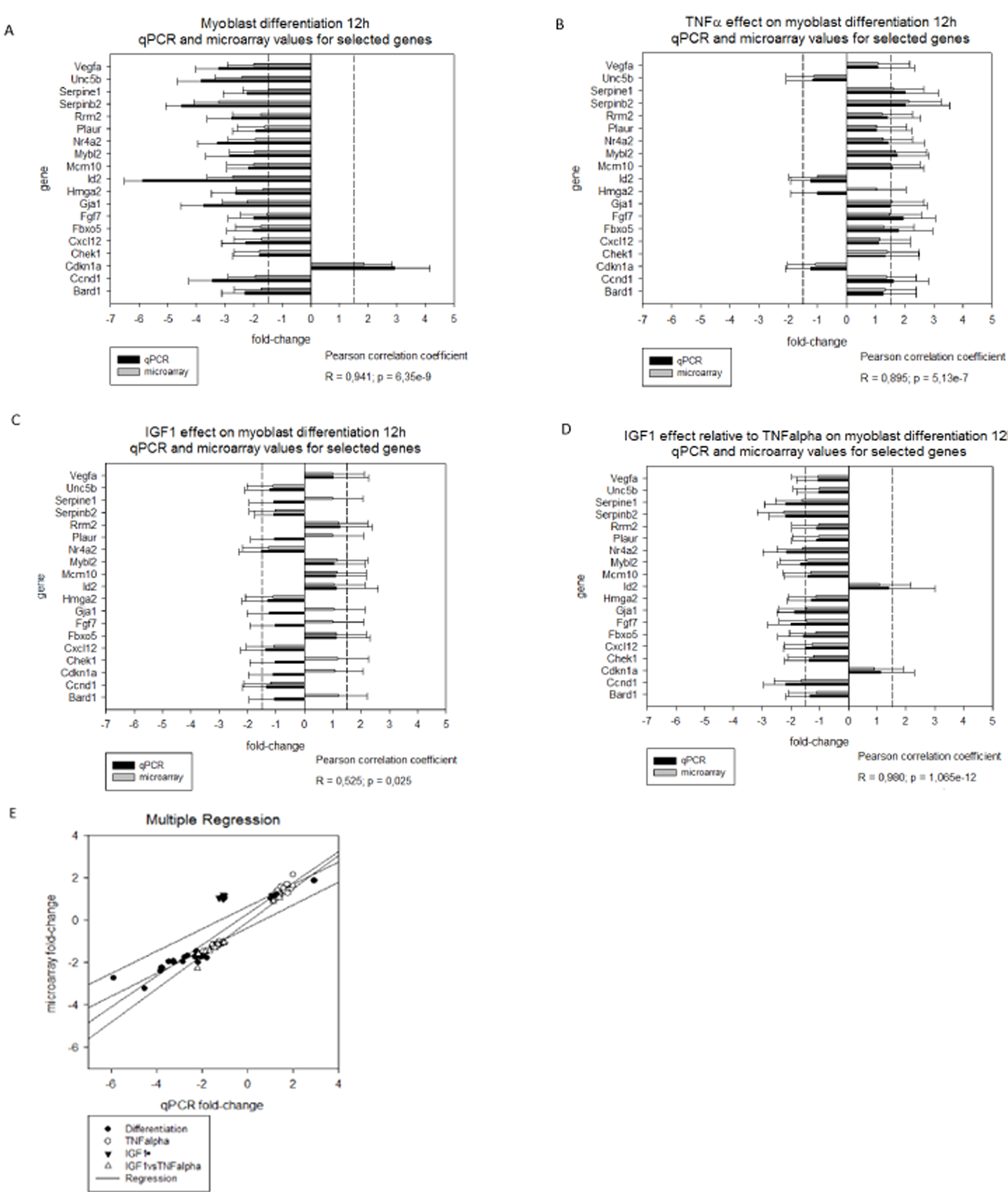

Supplement: S3 Fig — Relative fold-changes of Affymetrix gene expression profiling results and individual qPCR analysis results after 12 h of treatment are depicted for the effect of (A) differentiation, (B) TNF-α treatment, (C) IGF1 treatment, and (D) IGF1 treatment relative to TNF-α treatment. In each graph, the Pearson correlation coefficient R and the corresponding p values of microarray and qPCR results are shown. (E) Multiple regression analysis for relative expression values of microarray and qPCR analysis. (TIF) [file pone.0139520.s003.tif]

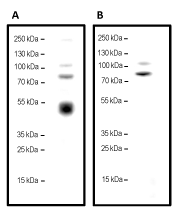

Supplement: S4 Fig — Western blot of Emi1/Fbxo5. (A) Emi 1 protein was detected at 46 kDa. However, we detected two unexpected bands at approximately 80 kDa and 110 kDa. (B) The Emi1-antibody was incubated with a 5-fold molar excess of Emi1 epitope rather than antibody. Specificity of the band at 46 kDa was confirmed as the band disappeared in contrary to the nonspecific bands at 80 kDa and 110 kDa. (TIF) [file pone.0139520.s004.tif]
